# Emergence and evolution of the glycoprotein hormone and neurotrophin gene families in vertebrates

**DOI:** 10.1186/1471-2148-11-332

**Published:** 2011-11-15

**Authors:** Sandra Dos Santos, Sylvie Mazan, Byrappa Venkatesh, Joëlle Cohen-Tannoudji, Bruno Quérat

**Affiliations:** 1 Univ. Paris Diderot, Sorbonne Paris Cité, Unité Biologie Fonctionnelle et Adaptative (BFA), EAC4413 CNRS, F-75013 Paris, France; 2 Marine Biology Research Division, Scripps Institution of Oceanography, University of California San Diego, La Jolla, CA 92093-0202, USA; 3 Développement et évolution des vertébrés, UMR 7150 CNRS - Université Pierre et Marie Curie Paris 6, Université Européenne de Bretagne, Station Biologique, Place Georges Teissier, 29682 Roscoff, France; 4 Institute of Molecular and Cell Biology, A*STAR (Agency for Science, Technology and Research), Biopolis, 138673, Singapore

## Abstract

**Background:**

The three vertebrate pituitary glycoprotein hormones (GPH) are heterodimers of a common α and a specific β subunit. In human, they are located on different chromosomes but in a similar genomic environment. We took advantage of the availability of genomic and EST data from two cartilaginous fish species as well as from two lamprey species to identify their repertoire of neurotrophin, lin7 and KCNA gene family members which are in the close environment of *gphβ*. *Gphα *and *gphβ *are absent outside vertebrates but are related to two genes present in both protostomes and deuterostomes that were named *gpa2 *and *gpb5*. Genomic organization and functional characteristics of their protein products suggested that *gphα *and *gphβ *might have been generated concomitantly by a duplication of *gpa2 *and *gpb5 *just prior to the radiation of vertebrates. To have a better insight into this process we used new genomic resources and tools to characterize the ancestral environment before the duplication occurred.

**Results:**

An almost similar repertoire of genes was characterized in cartilaginous fishes as in tetrapods. Data in lampreys are either incomplete or the result of specific duplications and/or deletions but a scenario for the evolution of this genomic environment in vertebrates could be proposed. A number of genes were identified in the amphioxus genome that helped in reconstructing the ancestral environment of *gpa2 *and *gpb5 *and in describing the evolution of this environment in vertebrates.

**Conclusion:**

Our model suggests that vertebrate *gphα *and *gphβ *were generated by a specific local duplication of the ancestral forms of *gpa2 *and *gpb5*, followed by a translocation of *gphβ *to a new environment whereas *gphα *was retained in the *gpa2*-*gpb5 *locus. The two rounds of whole genome duplication that occurred early in the evolution of vertebrates generated four paralogues of each gene but secondary gene losses or lineage specific duplications together with genomic rearrangements have resulted in the present organization of these genes, which differs between vertebrate lineages.

## Background

The pituitary gonadotropins, luteinizing hormone (LH) and follicle-stimulating hormone (FSH), together with the pituitary thyrotropin (TSH) are the core members of the glycoprotein hormone (GPH) family. These hormones are heterodimers composed of a common α (GPHα) and a β subunit that confers biological specificity. Characterizations of the GPH family in a wide range of osteichthyes have clearly shown that the common α subunit and the three GPHβ subunit lineages were already present before the split between actinopterygians (the branch leading to teleosts) and sarcopterygians (including tetrapods) [1]. An unambiguous GPHα as well as two GPHβ subunits were also isolated from a chondrichthyan, the small spotted catshark *Scyliorhinus canicula *[2]. The latter appeared related in sequence to LHβ and FSHβ, the presence of a third, TSHβ-related form, remaining an unanswered issue. Finally, analyses of GPH in cyclostomes (extant jawless vertebrates or agnatha) have provided evidence for a unique gonadotropin (GtH), with the isolation of a single GPHβ-related cDNA in the lamprey *Petromyzon marinus *[3] and of one α and a single GPHβ-related cDNA in the hagfish *Paramyxine atami *[4].

To gain insight into their evolutionary process, the genomic environment of these genes needs to be further explored. Among the genes in synteny with the *gph*, the neurotrophin (NT) genes have already been submitted to an evolutionary investigation. NT are homodimeric growth factors that play important functions in neuronal development and survival [5]. There are four NT types in tetrapods: nerve growth factor (NGF), brain derived neurotrophic factor (BDNF), neurotrophin 3 (NTF3) and neurotrophin 4 (NTF4) (also named NTF5 or NTF6). The same set of NT is present in teleosts with an additional, NGF-related NT (NTF7) [6]. Two out of the four NT were evidenced from chondrichthyes, one characterized as BDNF, the other one being closer to NTF3 [7] and a single NT (NT1) has been isolated to date from the lamprey *Lampetra fluviatilis *[8]. Interestingly, three NT genes appeared located in the human genome in the direct vicinity of the three GPHβ subunit genes which prompted the authors to propose that GPHβ and NT genes may derive from successive duplications from a genomic segment bearing the ancestral NT and GPHβ subunit genes [8,9]. It was tempting to assume that these duplications have taken place during the two rounds of whole genome duplication (WGD) that occurred between the divergence of urochordates (the sister group of vertebrates) and the radiation of gnathostomes (see [10] and references herein).

In order to test this hypothesis, we used BLAST analysis to exhaustively search the elephant shark *Callorhinchus milii *[11] and the sea lamprey *Petromyzon marinus *genomic sequences for the presence of genes that are in the close genomic environment of *gphβ *and *nt*. These genomic data where enriched with data from an EST analysis from another cartilaginous fish, *Scyliorhinus canicula *and from another cyclostome, *Lampetra fluviatilis*. When necessary, PCR amplifications from genomic DNA were used to complete the data.

Unlike NT, which are present in both deuterostome and protostome representatives [12], canonical GPHα, LHβ, FSHβ and TSHβ cannot be identified outside vertebrates [13]. However, human genome survey unexpectedly pointed to the presence of two proteins sharing common structural characteristics with GPHα and β subunits, which were consequently termed glycoprotein α-2 (GPA2) and glycoprotein β-5 *(*GPB5) [14]. In addition to their structural likeness, the recombinant proteins were shown to form a heterodimer that was able to bind and activate a GPH receptor (GPHR), namely the TSHR, both *in vitro *[15-17] and *in vivo *[16], giving an additional argument in support to a parental relationship between GPA2 and GPB5 and the GPHα and β subunits. Further genome explorations revealed the presence of *gpa2 *and *gpb5*-related genes (hereafter named *gpa *and *gpb*, respectively, when dealing with protostomes and pre-vertebrate deuterostomes) in most bilaterian groups [15,18-22]. Remarkably, the recombinant GPA and GPB proteins from the fruit fly were also shown to form a heterodimer able to activate the fruit fly receptor homologous to the vertebrate GPHR [18]. Interestingly, in *Nasonia vitripennis *at least, a hymenoptera in which *gpa *and *gpb *are not found in the genome [22,23], the GPHR-related receptor gene is also missing [23]. The same observation was made in the leech *Helobdella robusta *[24], which suggests that the presence of the receptor is strongly linked to the presence of this heterodimeric potential ligand.

The GPHβ subunits have no known role either as monomer or homodimer except for the β subunit of the human chorionic gonadotropin, which is believed to interfere with the TGFβ apoptotic action in certain cancer cell lines [25]. All vertebrate GPHβ subunits share the α subunit as an exclusive dimerisation partner. It is then highly probable that the ancestral β subunit also had the α subunit as a partner. The GPHα and ancestral GPHβ must have then been generated concomitantly. When present, *gpa *and *gpb *are localized next to each other in most invertebrate genomes including the urochordate *Ciona*. It is also the case in teleosts where *gpa2 *and one of the two paralogous *gpb5 *forms have kept this organization [22]. Taken together, these data strongly suggest that *gphα *together with the ancestral *gphβ*, as well as *gpa2 *and *gpb5 *originated from the duplication of a locus containing the closely located *gpa *and *gpb *and that this event took place sometime after the emergence of urochordates.

In order to fill the gap between the *gph *subunit genes and their molecular ancestors, we took advantage of the growing availability of genomic data in chordates to extensively characterize the *gpa-gpb *environment in amphioxus as well as the vertebrate environments of *gphα *and *gphβ *subunits and of *gpa2 *and *gpb5*. By comparing the syntenic relationships of these environments, we were able to reconstitute the ancestral chordate and vertebrate *gpa/gpb-*related gene environment and to propose a model of evolution of these genes during vertebrate radiation, involving gene duplications, transpositions and secondary losses.

## Methods

### PCR amplification and molecular biology

Elephant shark *Callorhinchus milii*, smaller spotted catshark *Scyliorhinus canicula*, sea lamprey *Petromyzon marinus *or European river lamprey *Lampetra fluviatilis *genomic DNA or cDNA were used for PCR amplifications. *Callorhinchus milii *pituitary RACE-ready cDNA were synthesized from total RNA using SMART RACE cDNA Amplification Kit (Clontech, USA) according to manufacturer's protocol.

The list of oligonucleotide primers used for this study is available on request: they usually are 20-23 nucleotides long with a melting temperature (Tm) ranging from 60 to 68°C. Reactions were realized in a volume of 25 *μ*l containing 10 ng of genomic DNA (or of a 1/1000th dilution of a primary PCR reaction or of a purified fragment), 0.5 units of GoTaq DNA polymerase (Eurogentec, Saraing, Belgium) with its appropriate 1× buffer supplemented with 0.25 mg/ml bovine serum albumin (when capillary tubes were used), 2.5 mM MgCl2, 0.2 mM dNTP and 0.2 *μ*M of each primer. PCR reactions were either run on a 1605 Rapid Cycler (Idaho Technology, Idaho falls, ID) with a denaturation step of 1 min at 94°C followed by 35-40 cycles of 10 sec at 94°C, 10 sec at 5°C under the Tm of the oligonucleotide with the lowest Tm and 30 sec - 1 min at 72°C or on a BioRad C1000 thermal cycler using the same parameters except that 30 sec steps were used rather than 10 sec's. A 3' tailing was achieved by a 30 min additional incubation step at 72°C when subcloning of amplified fragments were to be performed. Amplified fragments were eluted from 1× TAE (40 mM Tris, 2 mM acetic acid, 1 mM EDTA) buffered agarose gel using MinElute Qiagen extraction kit (Qiagen AS, Oslo, Norway). They were then either used as template for nested PCR or subcloned into pGEM-T easy vector (Promega Corporation, Madison, WI) or pCRII-TOPO vector (Invitrogen Corporation, Carlsbad, Ca) and sequenced (value read sequencing at MWG Biotech, Ebersberg, Germany).

All sequences of *C. milii *have been submitted to GenBank (accession numbers HQ174782 to HQ174794). The EST sequences were obtained through high throughput sequencing of several tissue or stage specific cDNA libraries: a *Lampetra fluviatilis *embryonic-prolarval (stages 20-26) cDNA library (EMBL:FR693754], a *Petromyzon marinus *larval head cDNA library [26] (EMBL:FR693760; BN001520-BN001524) and a *Scyliorhinus canicula *embryonic cDNA library [27] (EMBL::FR693755-FR693759).

### Database searches

Most sequences were obtained by BLAST analyses [28] on public databases on the NCBI website (http://blast.ncbi.nlm.nih.gov./Blast.cgi) using nucleotide or protein sequences as query depending on the phylogenetic distance between the query and the target database. The elephant shark *Callorhinchus milii *and lamprey *Petromyzon marinus *sequences were first BLAST searched on Trace archives of the whole genome shotgun (WGS) sequence databases for these species on NCBI or on the Contig reconstructions available at the dedicated server (http://esharkgenome.imcb.a-star.edu.sg/) for *Callorhinchus *genome or at the Genome Sequencing Center website of the Washington University in Saint Louis for *Petromyzon *genome (http://genome.wustl.edu/tools/blast/Petromyzon_marinus-3.0).

### Genomic clustering and synteny analysis

Genomic environments were analyzed using the NCBI Map viewer website (http://www.ncbi.nlm.nih.gov/mapview/) for human (v36), zebrafish (*Danio rerio: *Zv9*)*, chicken (*Gallus gallus: *built 2.1) and lizard (*Anolis carolinensis*: built 1.1) genomes, Ensembl website (http://genome.ucsc.edu/cgi-bin/hgGateway) for lamprey (*Petromyzon marinus*: assembly WUSTL v3.0) genome and the JGI websites (http://genome.jgi-psf.org/) for amphioxus (*Branchiostoma floridae *assembly v1.0) and the sea squirt (*Ciona intestinalis *assembly v2.0) genomes.

The paralogous gene sets were determined and refined by comparisons between the amphioxus and vertebrate genomes in a procedure including several successive steps. We first used the Genomicus [29] online genomic analysis tool (http://www.dyogen.ens.fr/genomicus-57.01/cgi-bin/search.pl) to trace back amphioxus (*Branchiostoma floridae *assembly version 2) homologues of genes found in the direct vicinity of *gpa2*, *gpb5 *and GPH subunit genes in human, chicken or zebrafish genomes. Analysis of their location showed that a large number of them were concentrated on a limited number of scaffolds of variable length in amphioxus. In a reverse approach and still using Genomicus, the second step consisted in determining the location in human genome of the homologues of all amphioxus genes carried by those of the scaffolds containing the higher concentration of homologues. The individual members of the corresponding human gene families appeared to be distributed among definite areas with synteny relationship with the amphioxus scaffolds. New areas were therefore identified that contained paralogues of genes located in the genomic environment of *gph *or *gpa/gpb *related genes. In a third step, we then systematically determined the location in the human genome of the paralogues of the genes present in each of these areas. Paralogous relationships at the vertebrate level were obtained *a priori *on the Ensembl server (http://www.ensembl.org/index.html). When the level of paralogy was not clearly determined, BLAST analyses were performed using human sequences against the amphioxus genome. If some related human sequences matched a single amphioxus gene, the target amphioxus gene was reverse-blasted against the human genome to verify that the initial human query genes were indeed the true homologues (*ie *they obtained the higher blast hit values). At the end of this third step, we were then able to better determine the size and gene composition of a number of paralogous regions in the human genome. In a fourth step and in order to ensure their grouping into four paralogous gene sets (*ie*, tetra-paralogons) the location of orthologues of representative (with regard to their position) genes identified in these paralogous regions were determined in chicken and lizard genomes on the Ensembl server. In a final step, we identified all the amphioxus homologues to the human genes that constituted the tetra-paralogons. This was done by using the search procedure on the JGI website (http://genome.jgi-psf.org/cgi-bin/searchGM?db=Brafl1) of *Branchiostoma floridae *genomic version 1 then by checking that the best hit in human of the obtained gene model indeed corresponded to the gene with which the homologous relationship was looked for. The scaffold position was then obtained by blasting the gene model of version 1 on the amphioxus genome version 2 which was the version used for genome comparisons.

### Phylogenetic reconstructions

Phylogenetic analyses were performed on protein sequences aligned using Se-AL software (http://tree.bio.ed.ac.uk/software/seal) either by using a maximum likelihood method with PhyML 3.0 software [30] with WAG as substitution model and the default settings on the web server phylogny.fr (http://www.phylogeny.fr/version2_cgi/phylogeny.cgi) or by a maximum parsimony method with PAUP version 4.1beta (Phylogenetic Analysis Using Parsimony [31]). The robustness of the reconstructions was estimated by bootstrapping using (100 replicates for PhyML, 1000 for PAUP).

## Results and Discussion

### Conserved gene organization of GPHβ subunit gene loci in teleosts and tetrapods

**Figure 1:**
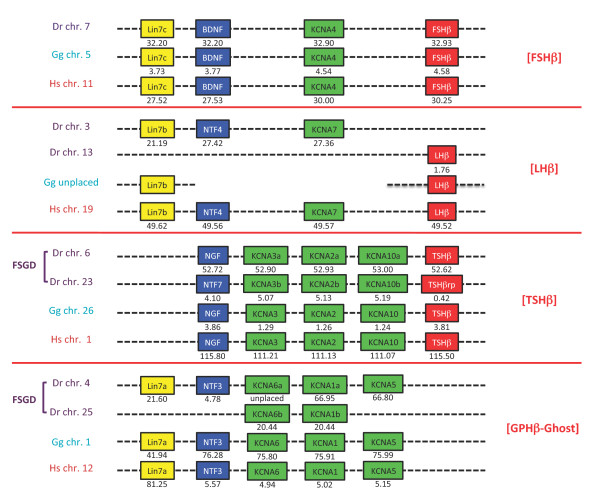
**Comparative genomic environment of *gphβ *subunit genes in representative vertebrates**. Human (Hs), chicken (Gg) and zebrafish (Dr) *gphβ *(*lhβ, fshβ *and *tshβ*) environment ([GPHβ]) are shown with their coordinates in megabase pairs from the p end of the relevant chromosome (chr.). [LHβ] is not found in chicken genomic databases but the existence of at least *lhβ *and *lin7b *is known from their cDNA product. A fish-specific genomic duplication (FSGD) generated specific duplicates in zebrafish. A paralogous environment devoid of any *gphβ *subunit gene is named [GPHβ-Ghost].

As initially shown by Hallböök and his collaborators [8,9], the three human GPHβ subunit genes lie on different chromosomes but share a synteny to NT genes, *lhβ, fshβ *and *tshβ *being located in the close proximity of *ntf4*, *bdnf *and *ngf *on human chromosome (chr.) 19, 11 and 1, respectively (Figure 1). A fourth NT gene (*ntf3*) is also present on human chr. 12 but no trace of another *gphβ *has been found in its surroundings. In order to assess the conservation of this synteny relationship in gnathostomes, we first analyzed the *gphβ *gene environment in zebrafish, chicken and human genomes (Figure 1). The association between *gphβ *and *nt *genes observed in human was found to be conserved in all three species, except for the *lhβ *subunit. In zebrafish, this subunit is located on chromosome 13 while *ntf4 *lies on chromosome 3. In chicken, although LHβ subunit cDNA was characterized years ago [32] and also cloned in more recent studies (GenBank:HQ872606), the corresponding gene remained undetectable by BLAST analysis in the current chicken genomic database. A further search of two other bird genome sequences (zebra finch *Taeniopygia guttata *and turkey *Meleagris gallopavo*) led us to the same disappointing result although LHβ cDNA (GenBank:L35519.1) has also been cloned from turkey. This will be further discussed below. The zebrafish *ngf/tshβ *environment on chr. 6 was found to be duplicated on chr. 23, which contains a teleost-specific *ngf *paralogue termed *ntf7 *[6], in the vicinity of a *tshβ*-related gene (*tshβrp: *GenBank:XM_001341527.1). The sequence of the latter was poorly conserved but still presents characteristics of a cystine-knot protein and contains a potential glycosylation site in a conserved position with other TSHβ subunits (Additional file 1: GPHβ subunit sequences) suggesting that it must be able to form a heterodimer with an α subunit and play a physiological role. Three additional potential glycosylation sites are even present, one in the same position as in FSHβ or LHβ, a second one, just after the 10^th ^cysteine residue and a third one, less likely, just in-between cysteine residues 8 and 9. This duplication is related to the large-scale genomic duplication (Fish Specific Genomic Duplication or FSGD) known to have occurred early during the evolution of teleosts [33,34].

Searches for additional conserved synteny with the *gphβ/nt *environments (named after their *gphβ *type put in brackets, the *ntf3 *region devoid of any *gphβ *being named [GPHβ-Ghost]) showed that they also include the members of the Kv1 family of shaker-related voltage-gated potassium channels (KCNA) and all *lin7 *gene family members (Figure 1). These gene families were particularly important for our studies on the evolution of the *gphβ *subunit environment because *kcna4 *and *kcna7 *are next to *fshβ *on human chr. 11 and to *ntf4 *on chr. 19, respectively and because *lin7c and lin7b *are next to *bdnf *on chr. 11 and to *tshβ *on chr. 1, respectively, suggesting that the ancestors of all four gene families must have been neighbors in a tight locus in an ancestral genome. As expected, *lin7a *is conserved in [GPHβ-Ghost], *lin7b *in [LHβ] and *lin7c *in [FSHβ] (Figure 1). One single *kcna *gene is found conserved in [FSHβ] and [LHβ], whereas 3 sets of genes are clustered in [TSHβ] and [GPHβ-Ghost]. From the duplicated [GPHβ-Ghost] in zebrafish, only 2 *kcna *genes were maintained. As previously shown [35] the duplicated *kcna5 *gene was lost, so were the duplicated *nt *and *lin7 *genes. As for *lhβ*, no *lin7b*, *ntf4*, *kcna7 *sequences could be identified in the chicken genome (while a Lin7b cDNA is known to exist: [GenBank:CN234764.1]). Such discrepancies between cDNA and genomic data have been reported for a high number of chicken or turkey genes, whose orthologues are located on human chr. 19q [36-38]. However, a locus including *lin7b *[Ensembl:ENSACAG00000013247], *kcna7 *[Ensembl:ENSACAT00000013329] and *lhβ *[Ensembl:ENSACAG00000013327] is found in the lizard *Anolis carolinensis *(chr. 6 at 80 Mb on AnoCar2.0 at Ensembl, see below), indicating that they were also grouped in the last common ancestor of diapsides.

Taken together, these data convincingly confirm the hypothesis [8] that expansion of the NT and GPHβ subunit genes derived from duplications of an ancestral locus that also contained a Lin7 gene and at least one KCNA gene. These duplications must have taken place before the split between tetrapod and teleost ancestors.

### Characterization of the GPHβ, NT, Lin7, KCNA gene repertoire in chondrichthyes

In order to better assess the timing of the duplication events giving rise to the *gphβ-nt-lin7*-*kcna *regions, we conducted a systematic search for these genes in two chondrichthyes, the elephant shark *C. milii *and the dogfish *S. canicula*.

*GPHβ*. BLAST analysis of elephant shark genome survey sequence allowed us to identify the first and second coding exons of *fshβ *on separate genomic fragments and the first exon of *tshβ *(Additional file 1). No BLAST hit was obtained for *lhβ *possibly due to the low, 1.4 × coverage of the elephant shark genome since cDNA encoding LHβ and FSHβ subunits were cloned from another cartilaginous fish species, *Scyliorhinus canicula *[2]. However, genomic PCR using primers for the two elephant shark *fsh *exons confirmed that the two exon sequences corresponded to the same gene. The sequence of the first exon of *tshβ *was used to re-BLAST a *Callorhinchus *pituitary cDNA library and two different TSHβ cDNA were identified, one of which matched the first exon sequence (Additional file 1). These results, together with those previously reported [2], definitively demonstrated the presence of the three *gphβ *subunit gene lineages, *lhβ, fshβ *and *tshβ*, in chondrichthyes. The existence of two *tshβ *subunit genes in *Callorhinchus *is discussed below.

*NT genes*. Three complete NT coding sequences and two additional 5' and 3' non-overlapping fragments were identified by an initial BLAST search on elephant shark genomic data. The first three sequences were confidently identified as NTF4, BDNF and NGF by sequence similarity (Additional file 2: Neurotrophin sequences). Indeed, the N-terminal ends of NT proteins are quite specific and difficult to align between the different types whereas the cystine-knot coding regions are very conserved, hence not very helpful for reliable phylogenetic analyses. The 5' fragment was clearly part of the NTF3 as confirmed by its identity with the full-length sequence of the dogfish NTF3 cDNA obtained by BLAST search on the *Scyliorhinus *cDNA library. PCR was used to extend the 5' NTF3 fragment toward the 3' end and the newly generated sequence complemented the end of another genomic segment of the database, thus completing the coding region of elephant shark NTF3. Similarly, PCR experiment using degenerate oligonucleotides allowed us to extend the other, 3' fragment, toward the 5' end and to obtain a sequence that was used to BLAST search the genomic data library where an overlapping 5' fragment was found. The reconstituted full length fifth NT encoding sequence was checked by PCR cloning on shark genomic DNA. This fifth NT presented 61.5% identity with NTF4 at the nucleotide level but part of its N-terminal amino acid sequence was totally different. When aligned with NTF4, a deletion of two nucleotides was evidenced in the N-terminal part of the sequence, inducing a frame shift that was corrected by a single additional nucleotide deletion 78 nucleotides downstream, resuming the original reading frame (Additional file 3: Elephant shark NTF4 and NTF4rp). This sequence was thus named NTF4rp. NTF4 but not NTF4rp was identified in addition to NTF3 from the *Scyliorhinus *cDNA library (Additional file 2). NTF4, NTF4rp and NGF are described here for the first time in chondrichthyes.

**Figure 2:**
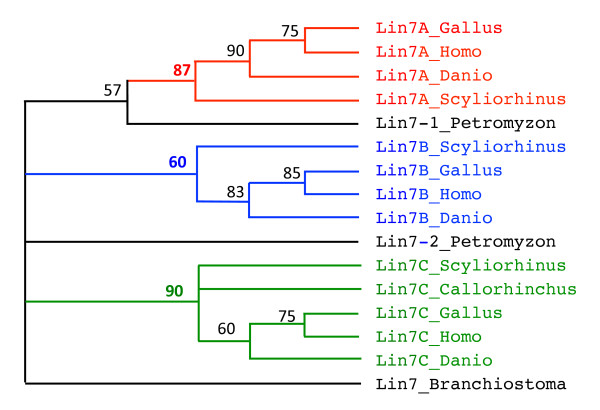
**Phylogeny of Lin7b sequences**. Phylogenetic reconstruction of chicken (*Gallus*), human (*Homo*), zebrafish (*Danio*) dogfish (*Scyliorhinus*), lamprey (*Petromyzon*) and amphioxus (*Branchiostoma*) lin7-related sequences was inferred using the maximum parsimony method (heuristic search). Bootstrapping (values at the nodes) was used over 1000 replicates. Lin7-A, -B and -C are clustered into monophyletic groups (bootstrap value in bold) that are highlighted in different colors.

**Figure 3:**
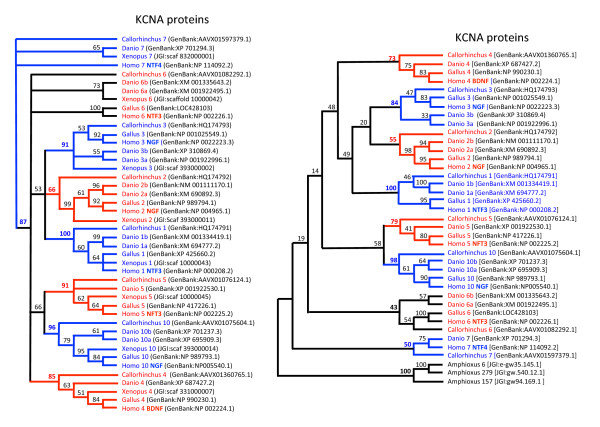
**Phylogeny of KCNA sequences**. Left panel: *Xenopus*, chicken (*Gallus*), zebrafish (*Danio*), elephant shark (*Callorhinchus*) and human (*Homo*) KCNA sequences. Phylogenetic reconstructions were inferred using the maximum parsimony method (heuristic search). Bootstrapping (values at the nodes) was used over 1000 replicates. The KCNA type is indicated by the number following the species name. The neurotrophin-type to which they are neighbor in human genomes is indicated in bold with the human sequence reference. All but KCNA6 were included into monophyletic groups (highlighted by alternate colors) that were supported by bootstrap values of 50% and over (values in bold). Right panel: amphioxus sequences are substituted to *Xenopus *sequences. None of the amphioxus sequence is closer to either vertebrate KCNA type. Lower bootstrap values were obtained when amphioxus sequences were included because only truncated sequences could reliably be aligned.

*LIN7 and KCNA*. BLAST searches for *lin7-*related genes in *S. canicula *databases led to the identification of three different ESTs corresponding to *lin7a*, *lin7b *and *lin7c *and provided no evidence for an additional member of the *lin7 *family (Figure 2, Additional file 4: Lin7 sequences). Similarly, four different genomic fragments were found in *C. milii *draft genome sequence; the longest fragment encoded 4 of the 5 exons of what was clearly *lin7c*, two other genomic fragments included two additional 4^th ^exons, the last one representing an additional exon 3 (Additional file 4). This sampling was compatible with the existence of three *lin7*-related sequences. Finally, eight members of the *kcna *family were identified in the elephant shark genomic database. Six of them were almost complete (Additional file 5: Elephant shark KCNA sequences). Sequence alignment and phylogenetic analysis showed that each of them could confidently be linked to one member of the family (Figure 3).

### Identification of GPHβ, NT, Lin7 and KCNA genes in cyclostomes

*Petromyzon marinus *genomic DNA analysis revealed the presence of a single *gphβ *subunit gene. Like Sower and colleagues [39], we were unable to find a second *gphβ *sequence, either by BLAST search on genomic or EST data or by PCR on genomic DNA using primers designed from the most conserved regions. The GPHβ subunit was proposed to be related to FSHβ or LHβ and named GtHβ (for gonadotropin), based on sequence comparison and its ability to have its expression stimulated by GnRH [3]. Two GPH-type receptors were characterized in lamprey, GPHRI that binds GtH [40] and GPHRII, which appeared closer to a thyrotropin receptor [41]. It is then likely that a second, TSH-type ligand, also exists in lampreys. Three NT genes were characterized, referred to as NT1, NT2 and NTz (Additional file 2). NT1 had already been characterized from *Lampetra fluviatilis *[8]. *Nt2*, which we also characterized from *Lampetra fluviatilis *larval EST screened by BLAST, was on the same Contig1180 (42775nt long) as *gthβ*, suggesting that it may be related to *NTF4 *and/or *bdnf*. NT1 and NTz were quite dissimilar from each other and from the other NTs so that their assignment to either members of gnathostome NT family could not be determined. Two *lin7 *genes were found (Additional file 4) that could not be confidently assigned to any of the three Lin7a-b-c forms of gnathostomes by phylogenetic reconstruction (Figure. 2) and were thus named *lin7_1 *(EMBL:BN001523; genomic contig5027) and *lin7_2 *(EMBL:BN0015234; genomic contig34734 and several ESTs). Finally, a total of at least 12 members of KCNA-related sequences were also found (Additional file 6: Lamprey KCNA sequences). Their relationships with other vertebrate KCNA sequences could not be confidently determined.

### Evolution of the GPHβ subunit gene environment in vertebrates

**Figure 4:**
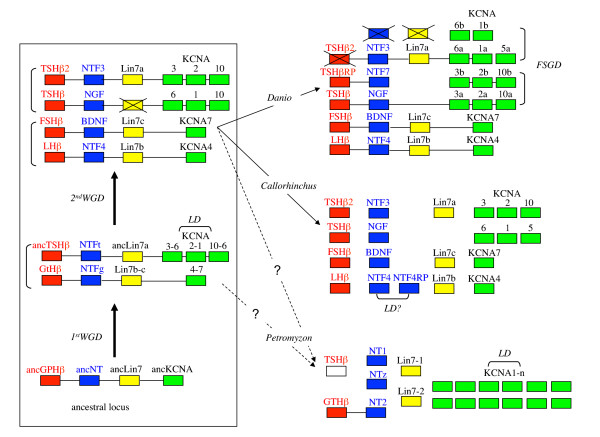
**Model for the evolution of *gphβ/nt *locus in vertebrates**. Left panel: evolution of the ancestral locus with the first and second whole genome duplication (WGD). Right panel: resulting known genomic loci in sea lamprey *Petromyzon marinus*, elephant shark *Callorhinchus milii *and zebrafish *Danio rerio*. It is still to be established whether cyclostomes have been submitted to the two rounds of genome duplication or not. Duplicate loci resulting from WGD or the Fish Specific Genome Duplication (FSGD) are indicated by vertical brackets whereas horizontal brackets represent local gene duplications (LD). Crossed boxes represent genes that have been lost after duplication. Genes demonstrated to be gathered in a definite locus are linked by a horizontal bar. The origin of *tshβ2 *in *Callorhinchus *is uncertain, either resulting from the second round of WGD or from a local duplication of *tshβ*. A *tshβ*-type subunit gene is expected in lamprey but has not been characterized yet and is represented as an empty box. NTFt and NTFg are given symbols for ancestral NTs associated with TSH and GTH, respectively.

Taken together, these data show that the duplication events leading to the presence of four sets of NT, three sets of GPHβ subunits and Lin7 genes, as well as of the eight KCNA forms, has taken place prior to the gnathostome radiation. This chronology of duplications as well as the conserved synteny observed for these genes in osteichthyes (actinopterygians plus sarcopterygians) is consistent with the hypothesis that the corresponding duplication events may have been part of the two rounds of WGD known to have taken place prior to the split between chondrichthyes and osteichthyes (Figure 4). As already proposed [35], local duplications of one of the *kcna *genes issued from the first round of WGD gave rise to three copies of the gene in one of the sister loci. It is still a matter of debate whether the second round of WGD occurred before or after the emergence of cyclostomes [42,43]. Depending on its timing, the cyclostomes would either have two or four sets of genes. However, our search led to the identification of one *gphβ *(but a *tshβ *is suspected to exist), two *lin7*, three *nt *and twelve or more *kcna *genes, which is inconsistent with either alternative and seems to indicate that some genomic data are missing (if the two WGD have already occurred) or that some of the genes were submitted to specific duplications in the lineage leading to *Petromyzon*. A higher quality genome coverage and assembly will be required to gain insight into the details of these genetic events. We also provide evidence for the occurrence of additional duplication events, giving rise to two *NTF4 *and two *tshβ*-related sequences in the elephant shark. Whether the latter two involved duplication of large chromosomal fragments remains an opened question in the absence of synteny arguments in chondrichthyes. In particular, an interesting possibility is that the second *tshβ *gene (*tshβ*2) may be part of the *ntf3-lin7a-kcna1-6-5-*containing region, as the fourth *gphβ *paralogue, possibly lost from the genome of osteichthyes (Figure 4). In other respects, of all the genomic environments generated by the fish-specific genome duplication it is noteworthy that only the one paraloguous to [TSHβ] remained unaltered in the zebrafish and still contains a *tshβrp *subunit, *ntf7 *and the three sets of *kcna*.

### Origin of the vertebrate GPHβ subunit gene environment

**Figure 5:**
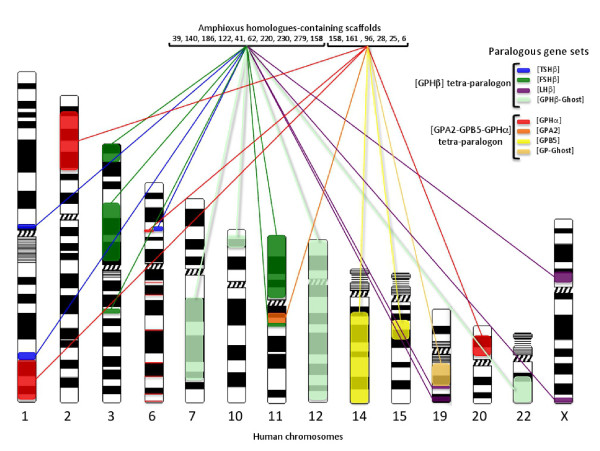
***Gph*-related gene-containing paralogous gene sets in human and amphioxus genomes**. This figure is a schematic representation of the data presented in Additional files 7 and 9 that shows the genomic distribution of the paralogous gene sets (tetra-paralogons) containing the *gph*-related genes in the human genome and lists the most important scaffolds (genome version 2) where the amphioxus homologues are located. The scaffold V2_158 contains genes that are homologous to genes belonging to one or the other tetra-paralogon.

**Figure 6:**
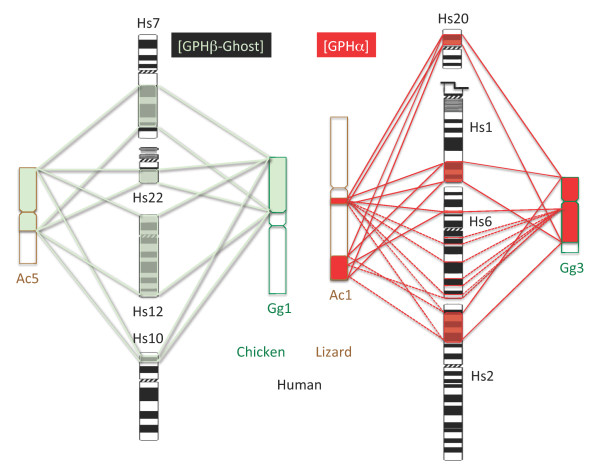
**[GPHα] and [GPHβ -ghost]-containing paralogous gene sets in human, chicken and lizard genomes**. Discrete gene locations are linked by dotted lines whereas more important loci are bordered by continuous lines (see Additional files 7 and 9 for details). The human chr. 1 was truncated for convenience. Hs: human; Gg: chicken; Ac: lizard.

If *gph*, *nt*, *lin7 *and *kcna *ancestors were neighbors in a tight locus in an ancestral genome (see above), numerous genomic rearrangements have occurred during the vertebrate radiation [44] and some descendant genes appear scattered in vertebrates (see on Figure 1 the position of *lin7a *relative to *ntf3 *or *kcna *genes on human chr. 12 and chicken chr. 1). In order to better characterize the ancestral composition of the GPHβ genomic environment, we needed to look at the genome of a species closer to the origin of vertebrates. The amphioxus genome was chosen over the highly rearranged genome of *Ciona*. We searched the amphioxus homologues of the genes found close to [LHβ], [FSHβ], [TSHβ] and [GPHβ-Ghost] in human, chicken and zebrafish genomes. Few amphioxus scaffolds contained homologues of genes from all four [GPHβ] environments. We then determined the location of the human homologues of all amphioxus genes carried by these scaffolds. Human homologous family members were distributed among definite genomic areas. The composition and boundaries of these areas were refined by determining the location of the paralogues of all their members, which were most often found within one or the other of these areas. At this stage, we had a number of scattered genomic areas, some of which including the previously characterized genomic environment of one or the other GPHβ gene, but their clustering into four paralogous sets of genes (ie tetra-paralogon) was not obvious. We therefore determined the position of zebrafish, chicken, and lizard orthologues of a number of human genes picked-up all along each of the regions. The zebrafish genome with the additional FSGD and numerous rearrangements turned out to be unsuitable for this task. However, some of the scattered regions in human were linked into a continuous segment in chicken and lizard genomes, allowing characterization of each of the four paralogous sets of genes (Figure 5, Table 1 and Additional file 7). For example, the paralogous gene set including [GPHβ-Ghost] appeared scattered among 4 chromosomes in human but was clustered on chicken chr. 1 and on lizard chr. 5 (Figure 6), indicating that its partitioning occurred sometime during the radiation of mammals. Also, the [TSHβ]-containing paralogous gene set that was divided between human chr. 1 and 6 was gathered on a definite segment of chr. 4 in lizard and of chr. 26 in chicken (Table 1 and Additional file 7). Almost none of the genes of the [LHβ]-containing paralogous gene set either from chr. 19 or chr. X was localized in the lizard genome or found in the chicken genome. However, as mentioned above about LHβ, and Lin7B, some of the products of these genes have been cloned in chicken (and other bird species): this is the case at least for GATA1 (GenBank:NM_205464), ALAS2 (GenBank:M24367), BCAP31 (Manchester chicken EST data base: ChEST959H20) and FLNA (GenBank:AB056474) that are located on human chr. X (see Additional file 7) and of GYS1 (GenBank:AB090806) the gene associated with *lhβ *and *lin7b *on human chr. 19. It has been hypothesized that the absence of these genes in the available genomic BAC libraries could result from bias in genomic cloning procedure [36] or, owing to the occurrence of specific repeats, to the difficulty in obtaining sequences from the smallest micro-chromosomes [37,38].

**Table 1:** *Gph*-related gene paralogous gene sets

Paralogous gene set	Human	Lizard	Chicken
**[LHβ]**	chr. 19: 49-51, 56-59 Mb	chr. 6: 79-81 Mb	not found
	
	chr. X: 47-56, 153-154 Mb	chr. 1: 205 Mb, chr. 2: 68-90 Mb	not found

	chr. 7: 77-140 Mb		
**[GPHβ-Ghost]**	chr. 10: 5-15 Mb	chr. 5: 0-100 Mb	chr. 1: 0-80 Mb
	chr. 12: 0-124 Mb		
	chr. 22: 33-51 Mb		

**[FSHβ]**	chr. 3: 0-15, 47-73, 125-129Mb	chr. 2: 147-191 Mb	chr. 12: 1-20 Mb
	
	chr. 11: 0-48, 68-70 Mb	chr. 1: 45-76 Mb	chr. 5: 0-23 Mb

**[TSHβ]**	chr. 1: 111-115/200-210 Mb	chr. 4: 105-140 Mb	chr. 26: 0-5 Mb
	chr. 6: 35-42 Mb		


	chr. 1: 210-245 Mb		
**[GPHα]**	chr. 2: 18-68 Mb	chr. 1: 127-143, 216-264 Mb	chr. 3: 2-80 Mb
	chr. 20: 6-25 Mb		
	chr. 6: 40-44/88/152/170 Mb		

**[GPB5]**	chr. 14: 27-106 Mb	chr. 1: 0-42 Mb	chr. 5: 26-62 Mb
	chr. 15: 33-43 Mb		

**[GPA2]**	chr. 11: 61-67 Mb	unplaced	not found

**[GP-Ghost]**	chr. 19: 36-49 Mb	unplaced	not found

Observed boundaries of *gph*-related gene-containing paralogous gene sets in human, lizard and chicken genomes are given in megabase pairs (Mb) on the relevant chromosome (chr.). Some of the paralogous gene sets are scattered over several chromosomes (See Additional files 7 and 9 for the gene composition of each paralogous gene sets).

The most compact paralogous gene set appeared to be the one including [TSHβ]. Despite its scattering into three separated regions in human, it was contained into a segment of 5 Mb in chicken and 35 Mb in the lizard genome. A total of 227 gene families were identified, 96 with paralogues associated with [LHβ], 130 with [TSHβ], 162 with [FSHβ] and 185 with [GPHβ-ghost]. Out of these 227 gene families, 199 were traced back to amphioxus (Additional file 7). Ten scaffolds contain 97 homologues of the vertebrate gene families, 20 of them being on scaffold V2_39, 13 on scaffold V2_158, 10 on scaffolds V2_140 and V2_186, 8 on scaffold V2_220, 8 on V2_122 and 7 on scaffolds V2_41, V2_62, V2_230 and V2_279.

Recently, the comparison of the chromosomal organization of amphioxus genome with those of different vertebrates led to a tentative reconstruction into 17 proto-chromosomes of the last common chordate ancestor genome [45] and to a partitioning of the human genome into segments that showed defined patterns of fourfold conserved synteny to those proto-chromosomes. Using a different approach, Nakatani and collaborators [44] proposed a reconstruction of 10 (out of *circa *10-13) proto-chromosomes of the ancestral vertebrate karyotype and identified their resulting paralogous linkage blocks in the human genome. In the human genome, the segmentations and boundaries of the four paralogous gene sets identified in this study most often matched those that were given as resulting from the partitioning of the ancestral vertebrate proto-chromosome (proto-chr.) D [44] (Additional file 8: Human [GPHβ] tetra-paralogon and ancestral chordate and vertebrate proto-chromosome relics). Only the segments on chr. X from the [LHβ]-containing paralogous set and the one on chr. 22 from the [GPHβ-ghost] paralogous gene set were not predicted to be derived from this ancestral vertebrate proto-chromosome. Conversely, we did not find any gene on chr. 20 that undoubtedly belonged to either paralogous groups included in the paralogon. It was more difficult to map the paralogous gene sets obtained in this study to those resulting from the ancestral chordate reconstruction by Putnam and collaborators [45] (Additional file 8). It looks like they would comprise all the segments derived from both chordate proto-chr. 13 and 14 as if these two proto-chromosomes were indeed combined into a single proto-chromosome.

A *lin7 *homologue is present in amphioxus on scaffold V2_124 (fgenesh2_pg.scaffold_629000011) but no other genes were found in this scaffold that had orthologues in *gphβ *subunits environment. The ancestral *lin7 *gene might thus have been translocated to the *gphβ *subunit locus after the divergence of the amphioxus lineage. An alternative hypothesis would be that *lin7 *had been specifically translocated away from a *nt*/*kcna *locus sometime in the branch leading to amphioxus. Two types of NT genes were found in several *ante*-vertebrate chordates but a single one had the same genomic organization (the entire coding region restricted to a single exon) as in vertebrates [12]. It is located on scaffold V2_138 in amphioxus genome and is surrounded by other genes that have orthologues in the *gphβ *genomic environments. Several amphioxus *kcna *genes might potentially be homologous to the ancestral vertebrate *kcna*: the first one [JGI:gw94.169.1 or GenBank:XM_002597415] is located on scaffold V2_157, close to homologues of genes that are in the [GPHβ] tetra-paralogon (Additional file 7); another one, [JGI:gw.540.12.1 or GenBank:XM_002586434] is on scaffold V2_279, also located next to genes related to those in *gphβ *environments; the third one, [JGI:e-gw35.145.1 or GenBank:XM_002613506] is on scaffold V2_6 that has numerous homologues in the paralogons corresponding to the GPA2/GPB5 environment (see below). All of these amphioxus *kcna *genes were encoded by a single exon. None of them appeared to be closer than the others to the vertebrate *kcna *genes by our phylogenetic analysis (Figure 3). They may then represent specific duplicates of *kcna *genes in the amphioxus lineage. Another possibility, that seems less likely, is that vertebrate KCNAs would have derived from two (or more) KCNAs at the ancestral vertebrate level. A comparative analysis of the KCNA environments on several chordate representatives would be necessary to address this question.

### Origin of GPA2 and GPB5 genomic environments

*Gpa2 *and *gpb5*-related genes have been reported in all vertebrate species analyzed so far with the exception of birds that seem to lack *gpa2 *[22]. Since GPH subunits are believed to originate from duplication of the ancestral form of these genes sometime before the radiation of vertebrates, it was important to reconstruct the evolutionary history of the environment. We followed a similar approach as for the *gphβ *subunits. This analysis allowed us to identify 4 paralogous sets of genes. The genomic environments were each part of one of *gpa2 *([GPA2]), *gpb5 *([GPB5]), *gphα *([GPHα]) were each part one of these paralogous gene sets. The fourth one did not contain any of the GPH subunit-related gene and was referred to as [GP-Ghost] (Figure 5, Additional file 9: [GPHα-GPA2-GPB5] tetra-paralogon). The most compact paralogous gene set was the [GPA2]-containing one and was condensed into 6 Mb of human chr. 11. Most of the corresponding genes were however not placed in the current assembled genome of the lizard and, as for the [LHβ]-containing paralogous gene set and likely because of the same difficulties, not found in the current version of the chicken and turkey genomes. The [GPHα]-containing paralogous gene set which was scattered among 4 different chromosomes in the human genome was found gathered onto chr. 3 in chicken and most genes were spread over two regions of chr. 1 in lizard (Figure 6, Table 1, Additional file 9). Similarly, the [GPB5]-containing paralogous gene set distributed between chr. 14 and 15 in human was restricted to a short region of chr. 5 in chicken and of chr. 1 in lizard.

In the human genome, a total of 197 gene families were identified, 171 associated with [GPHα], 164 with [GPB5], 71 with [GPA2] and 80 with [GP-Ghost]. Among them 187 were recovered in amphioxus, 127 distributed among 6 scaffolds with 38 of them being gathered on scaffold V2_6, 25 on V2_28 and V2_25, 14 on V2_161, 13 on V2_158 and 12 on V2_96 (Figure 5 and Additional file 9). It is to be noted that one scaffold, scaffold 158, had genes that mapped to the two tetra-paralogons with 13 genes involved in each of them. They mostly came from scaffold 10 of version 1 of the amphioxus genome with one end (up to 2.4 Mb) mapping to one tetra-paralogon and the other one to the other tetra-paralogon. One of the genes (e_gw.10.14.1: V2_158 EFEMP/FBLN), appeared to be homologous to *fbln*-*1 *and *2 *that are part of the [GPHβ] tetra-paralogon and also to *efemp-1 *and *2 *and *fbln5 *that are within [GPHα-GPA2-GPB5] tetra-paralogon.

Boundaries of these paralogous gene sets corresponded quite accurately to those of the segments described as resulting from the chordate proto-chr. 11 and from the vertebrate proto-chr. G (Additional file 8).

### Reconstruction of the *gpa/gpb *evolutionary history

The present study provides strong evidence that [GPHα], [GPA2] and [GPB5] environments derive from an ancestral vertebrate locus through the two rounds of WGD. The shared environment between *gphα *and *gpa2 *confirms the parental relationship previously suggested from their structural and biochemical properties. The shared environment between *gpa2 *and *gpb5 *confirms that ancestral *gpa *and *gpb *were on the same locus before the WGD strengthening the hypothesis that the ancestral *gphß *together with *gphα *and vertebrate *gpa2 *with *gpb5 *were created by the duplication of a locus containing the closely linked *gpa *and *gpb*. However, the fact that the [GPHβ] environments belong to a different tetra-paralogon, *ie *derive from a different ancestral vertebrate locus, indicates that the ancestral β subunit gene was transferred to a different locus before the two rounds of WGD occurred. Another gene, the one corresponding to e_gw.10.14.1 in amphioxus (V2_158 EFEMP/FBLN) might have been duplicated in the same duplication event as *gpa *and *gpb*. After the duplication of this gene prior to the WGD, one of the duplicated genes would have given rise to *efemp-1 *and *2 *and *fbln5 *that are part of the [GPHα-GPA2-GPB5] tetra-paralogon. Its sister gene would have been transferred to a locus close to the newly created ancestral *gphβ *and would have given rise to *fbln-1 *and *2 *that are part of the [GPHβ] tetra-paralogon. It is then likely that *gpa-gpb *were duplicated with a very limited number of genes among which could be the one homologous to V2_158 EFEMP/FBLN and that the ancestral *gphβ *was transferred *via *translocation or partition of the newly duplicated locus. Such a partition is indeed observed for the amphioxus locus present on V2_158 that has one part with homologues on the [GPHα-GPA2-GPB5] tetra-paralogon and the other on the [GPHβ] tetra-paralogon.

**Figure 7:**
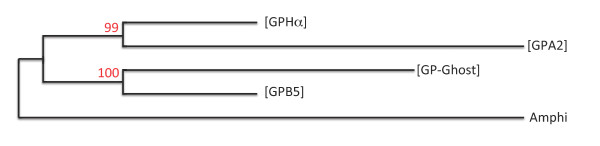
**Phylogenetic relationships between paralogous gene sets**. Maximum Likelihood inferred relationships between concatenated protein sequences (1959 informative positions retained) of human RTN, MAP3K, MAP4K, ACTN, MARK, SIPA, KLC and FOS paralogues from the four ([GPHα], [GPB5], [GPA2] and [GP_Ghost]) paralogous gene sets with their homologue in amphioxus (Amphi) as the outgroup. Bootstrap values (100 replicates) are indicated in red.

Earlier phylogenetic reconstructions indicated closer relationships between NTF4 and BDNF and between NTF3 and NGF [8,46]. Sequence analysis of the GPHβ subunits led us to the same conclusion that LHβ (which is on the same environment as NTF4) was closer to FSHβ (BDNF environment) than either of them was to TSHβ (NGF environment) [2]. The same conclusions about the relationships between these environments where drawn when the history of the KCNA family was explored [35]. The relationships between individual genes in these loci must apply for the entire corresponding paralogous gene sets. Accordingly, [LHβ] and [FSHβ]-containing paralogous gene sets on the one hand and [TSHβ] and [GPHß-Ghost] paralogous gene sets must have been generated from the duplication associated with the 2^nd ^WGD of two former paralogous gene sets which themselves derived from the ancestral vertebrate proto-chromosome with the 1^st ^WGD. In order to establish similar type of relationships within the *gpa2/gpb5 tetra-*paralogon, we performed a phylogenetic analysis of the concatenated human protein sequences of some of the genes that have all four paralogues in this paralogon (RTN(1-4), MAP3K(9-11), MAP4K(1-3/5), ACTN(1-4), MARK(1-4), SIPA(1/L1-3), KLC(1-4) and FOS(-/B/L1/L2)) using the amphioxus homologues as the outgroup. The paralogous relationship between [GPHα] and [GPA2] paralogous gene sets and between [GPB5] and [GP-Ghost] paralogous gene sets were supported by bootstrap values (Maximum likelihood, 100 replicates) of 100 and 99, respectively (Figure 7).

**Figure 8:**
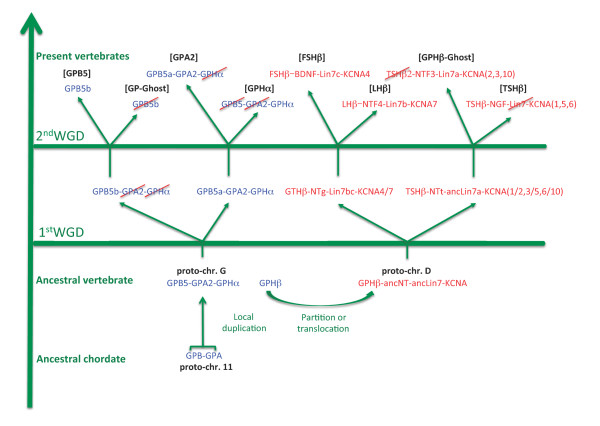
**Scenario for the evolution of the *gph-related gene *environments**. The chordate *gpa-gpb *locus was duplicated prior to the first round of genomic duplication and the newly created *gphβ *was transferred to vertebrate proto-chr D whereas *gpa2*, *gpb5 *and *gphα *localized on vertebrate proto-chr. G. Paralogues of *gpa2*-*gpb5-gphα *and *gphβ *environments were then created through two whole genome duplications (WGD). Genes that have been lost are crossed out in red. *Gpb5a *is still present in teleosts in [GPA2] environment but was lost in tetrapods (see Figure 9).

The evolutionary scenario of the *gpa2-gpb5 *environment in Figure 8 was determined so as to match the relationships between the paralogous gene sets. First, *gpa *and *gpb *were located next to each other on the predicted ancestral chordate proto-chr. 11. Ancestral forms of NT, Lin7 and KCNA must have been on a chordate composite proto-chromosome 13/14. The *gpa-gpb *close environment was then submitted to a specific, local duplication, generating *gphα *and ancestral *gphβ *subunits. This event took place between the radiation of urochordates (that do not have *gphα *and *gphβ *but do have a *gpa-gpb *locus) and the first round of genomic duplication. The environment must have then been split into two loci, one located on vertebrate proto-chr. D, close to the ancestral *lin7*, *kcna *and *nt *genes, and the other one on vertebrate proto-chr. G. This locus was then submitted to the two rounds of WGD to generate the four [GPHβ] environments as illustrated in more details in Figure 4.

**Figure 9:**
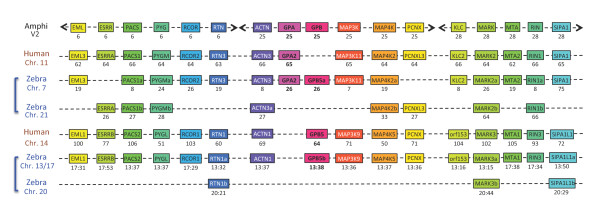
**Comparative genomic environment of *gpa2 and gpb5***. Genes are given with, underneath, their position in megabase pairs from the p end of each chromosome (chr.) in human and zebrafish (zebra) (see Additional file 10 for details and links to Ensembl website). Amphioxus (Amphi) homologues are given with the scaffold number (version 2) on which they are located. Names are after Ensembl in the human genome except for amphioxus homologues, which were reduced. Duplicated chromosome fragments in zebrafish are indicated with brackets. Due to rearrangements, the zebrafish *gpb5b *environment is scattered among chr. 13 and 17 whereas its FSGD driven duplicated environment is on chr. 20. Human GPB5 is orthologous to zebrafish GPB5b, whereas zebrafish GPB5a environment is orthologous to that of human GPA2.

After the first round of genomic duplication of the vertebrate proto-chr. G, one copy of both *gpa2 *and *gphα *was eliminated, leaving only one copy of each of them, whereas two copies of *gpb5 *were conserved, one of which still next to *gpa2*. The two loci were submitted to the second genomic duplication. One copy of *gap2*/*gpb5 *was lost on what was now the [GPHα]-containing paralogous gene set whereas the other copy of *gphα *was lost. A single copy of the *gpa2-gpb5 *linked genes was conserved in early vertebrates and is still present in teleosts (Figure 9 and Additional file 10: [GPA2] and [GPB5] environments in human and zebrafish). Indeed, most teleosts appear to have two copies of *gpb5*, one (*gpb5a*) next to *gpa2*, and the other one (*gpb5b*) in the same environment as tetrapod *gpb5*. In *Xenopus*, only one copy of *gpb5 *(e_gw1.491.6.1) is identified, located on a different scaffold as *gpa2 *(e_gw1.296.27.1). Both genes are in an environment syntenic with their mammalian counterparts (data not shown). It is worth noticing that two copies *of gpb5*, one complete ([EMBL:BN001271], [22]) and a fragment of the second encoding exon of another one (*Petromyzon *trace archives [GenBank:gnl|ti|1201055580, PMAC-akl10f02.b1]) showing 83% identity were detected in *Petromyzon *genomic data.

As shown in this study, the genes in the direct environment of amphioxus *gpa *and *gpb *do not map to the *gphβ *environments as it was postulated by Kubokawa and collaborators [21]. The genes used for their study were *actn, pcnx, map3k (9-11)*, and *map4k *whose homologues actually map to the [GPHα-GPA2-GPB5] tetra-paralogon (Additional file 9) and two other genes, *kirrel *and *pou2f *whose homologues are effectively located on human chr. 1 (156-172 Mb), chr. 11 (120-125 Mb) and chr. 19 (41-47 Mb) but outside the boundaries of the [GPHβ] tetra-paralogon. Only the *klhl28 *(chr. 11: 45 Mb) was actually within the boundaries of the [FSHβ-containing paralogous gene set but this gene does not seem to be paralogous at the vertebrate level to the two other mentioned *klhl20 *(chr. 1: 172 Mb) and *keap1 *(chr. 19: 10Mb) that, in any case, are both located outside the boundaries of any *gphβ *paralogous gene sets.

Our present study also differs from a previous report on the origin of neurotrophins by Hallböök and collaborators [9] in that they included in their neurotrophin-containing paralogous gene sets a number of genes that actually were part of the [GPHβ-GPA2-GPB5] tetra-paralogon. This was likely due to the juxtaposition in human of parts of the paralogous gene sets from the two tetra-paralogons on chr. 1 ([TSHβ: 200-210 Mb and [GPHα]: 210-245 Mb), chr. 19 ([LHβ:49-51 Mb and [GP-Ghost]: 36-49 Mb), and chr. 11 ([FSHβ:68-70 Mb and [GPA2]: 61-67 Mb) that could have been misleading at a time when most other vertebrate genomic data were not available. Indeed, these paralogous regions do not appear contiguous in lizard and chicken as exemplified by [TSHβ and [GPHα]-containing paralogous gene sets that are located on different chromosomes in lizard (chr. 4 and 1, respectively) and chicken (chr. 26 and 3, respectively) (Additional file 8).

## Conclusions

In this paper, we first investigated the repertoire for *gphβ*-related genes in cartilaginous fishes and lampreys, and some genes that were kept in their genomic environment in teleosts and tetrapods. This analysis shows that cartilaginous fishes are provided with the same set of GPHβ subunits as tetrapods. An additional *tshβ*-related gene is also found in the elephant shark *C. milii*. In addition to four NT genes, a fifth, *ntf4*-related gene is present in elephant shark. In lampreys, the only observed *gphβ*-related gene was the already known *gthβ*. In contrast, in addition to the previously characterized NT1 we were able to identify two additional NT, NT2 and NTz in *Petromyzon *but their sequences were too divergent for a reliable relationship with their tetrapod counterparts to be established. These results are consistent with an evolution driven by two successive WGD of a genomic region that contained a unique ancestral form of each of *gphβ, nt, lin7 *and *kcna *genes. To better characterize this ancestral environment, we conducted an analysis of the amphioxus genome and identified two sets of scaffolds containing genes homologous to those present in the environments of the vertebrate *gphβ *and in the environments of *gpa2, gpb5 *and *gphα*, respectively. By looking in vertebrate genomes for the location of all the genes in these scaffolds, we identified chromosomal segments in human, chicken and lizard that constituted two different tetra-paralogons. This allowed us to propose a scenario for the evolution of the *GPH and NT *gene families within their environment in vertebrates. Vertebrate *gphα *and *gphβ *were generated by a local duplication of the ancestral forms of *gpa2 *and *gpb5*, followed by a relocation of *gphβ *into a new environment whereas *gphα *was retained in the *gpa2-gpb5 *environment. Two rounds of WGD generated four paralogues of each gene in the two ancestral environments but secondary gene losses or lineage-specific duplications together with genomic rearrangements altered the present, species-specific organization of these genes.

## List of abbreviations

Most gene symbols are according to Ensembl nomenclature; BLAST: Basic local alignment search tool; GPH: Glycoprotein hormone; GPHR: GPH receptor; GtH: Gonadotropin hormone; NT: Neurotrophin; GPA2: Glycoprotein hormone alpha 2 (GPHA2 according to Ensembl); GPB5: Glycoprotein hormone beta 5 (GPHB5 according to Ensembl); GPA: Glycoprotein alpha (used to characterize pre-vertebrate GPA2); GPB: Glycoprotein beta (used to characterize pre-vertebrate GPB5); KCNA: Kv1 family of shaker-related voltage-gated potassium channels; WGD: Whole genome duplication; FSGD: Fish-specific; genome duplication

## Authors' contributions

SDS and BQ participated in the design of the study, carried out lab work and wrote the manuscript. SM and BV provided materials, participated in the lab work and in the writing. JCT participated in the writing. All authors read and approved the manuscript.

## Supplementary Material

Additional file 1 **GPHβ subunit sequences**. Amino acid sequences of elephant shark *Callorhinchus milii *FSHβ, TSHβ and TSHβ2 subunits [GenBank:HQ174783-HQ174785] characterized in this study and of zebrafish *Danio rerio *TSHβ [GenBank:AY135147] on chr. 6 and TSHβrp [GenBank:XM_001341527.1] on chr. 23. Cysteine residues involved in the cystine knot structure are in red. Potential N-liked glycosylation sites are in bold red. Click here for file

Additional file 2 **Neurotrophin sequences**. Amino-acid sequence of *Callorhinchus milii *(references from GeneBank), *Scyliorhinus canicula*, *Petromyzon marinus *and *Lampetra fluviatilis *(references from EMBL) NT encoding sequences characterized in this study aligned with *Lampetra *NT1 and human NT sequences for comparison (references from GeneBank). Cysteine residues involved in the cystine knot structure are in red. The amino acid sequence of the region of NTF4rp resulting from the shift in the open reading frame (see Additional file 3) is given in italic. Click here for file

Additional file 3 **Elephant shark NTF4 and NTF4rp**. Comparison of NTF4 [Genebank:HQ174787] and NTF4rp [Genbank:HQ174788] sequences from *Callorhinchus milii*. Nucleotide deletions causing a shift in the open reading frame are indicated by red dashes and resulting amino acids given in italic. Other differences are highlighted in grey. Click here for file

Additional file 4 **Lin7 sequences**. Alignment of Lin7-related encoding sequences of *Scyliorhinus canicula, Callorhinchus milii *(Callo) and *Petromyzon marinus *characterized in this study with the amphioxus *Branchiostoma floridae *homologue and sequences from human (*Homo*), chicken (*Gallus*) and zebrafish (*Danio*). Three paralogues are evidenced in *Scyliorhinus *and two in *Petromyzon*. References are from GeneBank except for *Scyliorhinus *sequences that are from EMBL. Positions specific to Lin7b sequences are highlighted in blue, those specific to Lin7a are in red and those to Lin7c are in green. Amino acids identical to *Branchiostoma *are given by dashes. Gaps are identified by dots. Unknown positions are indicated by interrogation marks. Exon boundaries are indicated in the bottom lines. Click here for file

Additional file 5 **Elephant shark KCNA sequences**. *Callorhinchus milii *KCNA sequences. Complete sequences were deposited at GenBank and are given with their accession number. Other, partial sequences are given with their reference from the genome survey sequence at GenBank. Sequences were numbered according to their phylogenetic relationship with other members of the vertebrate KCNA family (see Figure 3). Click here for file

Additional file 6 **Lamprey KCNA sequences**. *Petromyzon marinus *KCNA-related sequences extracted from their Contig sequences on the Washington University dedicated server (see Methods) and/or from the Traces archives deposited in GeneBank. The positions in the Contig or trace sequences in the sense or antisense strand (complement) are indicated. The underlined position corresponds to the first nucleotide of the 5' end ATG when appropriate. KCNA sequences were referenced by letters instead of numbers because their relationship with the different known KCNA-types could not be determined. Click here for file

Additional file 7 **[GPHβ] tetra-paralogon**. Listing of genes identified as constituting the paralogous gene sets comprising GPHβ subunit gene environments ([GPHβ]) in human with some representative orthologues in chicken (*Gallus gallus*) and lizard *(Anolis carolinensis) *genomes and the homologous sequences in amphioxus (*Branchiostoma floridae*). Amphioxus, chicken and lizard gene names are after the human orthologue names as in Ensembl release 62. Genes are given with their approximate location on the chromosome (chromosome number followed by the coordinate rounded up or down to the nearest in megabase pairs) or with their scaffold number (assembly version 2) for amphioxus gene. Each vertebrate gene has hyperlink to its dedicate Ensembl website and amphioxus gene to JGI website (assembly version 1). Genes that have paralogues in all four paralogous gene sets in the human genome are highlighted. nf: genes not found or for which the parental relationships did not appear reliable. Unplaced means that the position in the genome was not given on the Ensembl current release. * indicates chicken genes not found in the available genomic data (NCBI release 2.1) but for which a cDNA is known (see text). Genes may be sorted by columns in order to analyze specific paralogous gene sets. Click here for file

Additional file 8 **Human GPH-related gene paralogous gene sets and ancestral chordate and vertebrate proto-chromosome relics**. Observed boundaries of the paralogous gene sets comprising the GPH subunit-related genes in human compared to segments (S) or conserved vertebrate linkages (CVL) predicted to derive from the two whole genome duplication events of, respectively, ancestral chordate proto-chromosomes (proto-chr.) G and D, according to [45] and of ancestral vertebrate proto-chromosomes 11 and 13/14 according to [44]. [GPHβ] tetra-paralogon seems to correspond to the sum of the segments derived from proto-chr. 13 (given in red) and 14. Click here for file

Additional file 9 **[GPHα-GPA2-GPB5] tetra-paralogon**. Listing of genes identified as constituting the paralogous gene sets comprising [GPHα], [GPA2], [GPB5] and [GP-Ghost] environments with their amphioxus homologues. See legend of Additional file 7. Click here for file

Additional file 10 **[GPA2] and [GPB5] environments in human and zebrafish**. Listing of representative genes in the environment of human and zebrafish *gpa2 *and *gpb5 *with their amphioxus homologues. See legend of Additional file 7. Click here for file
