# Supplementary material for: Emergence and evolution of the glycoprotein hormone and neurotrophin gene families in vertebrates
Source: BMC Evol Biol. 2011 Nov 15;11:332. doi: 10.1186/1471-2148-11-332 (PMC3280201; doi:10.1186/1471-2148-11-332)
Supplement: Additional file 1 — GPHβ subunit sequences. Amino acid sequences of elephant shark Callorhinchus milii FSHβ, TSHβ and TSHβ2 subunits [GenBank:HQ174783-HQ174785] characterized in this study and of zebrafish Danio rerio TSHβ [GenBank:AY135147] on chr. 6 and TSHβrp [GenBank:XM_001341527.1] on chr. 23. Cysteine residues involved in the cystine knot structure are in red. Potential N-liked glycosylation sites are in bold red. [file 1471-2148-11-332-S1.PDF]

*Callorhinchus*_FSHβ HQ174783	MLLVMVNRISAAHGFLLILCWVSIHCQNNCQLTNITMAVEKEECGYCGNVNVSWCSGYCFTKD--PVFKERMASIYQYIC
*Callorhinchus*_TSHβ HQ174784	MNAMWLLPLVLCLSGSQIGFTCSLTRHVVYVEKEECSYCMAINTTVCAGYCMSRDVNIKTLLPKNALVQNVC
*Callorhinchus*_TSHβ2 HQ174785	MPMFCRMSSRLLLLILFLCGGRAHPYCSPSPYLQYLEQDQCEFCLVINTTICSGSCLTRDANVKRLLPKSALSQNIC
*Danio*_TSHβ AY135147	MSLLYVIGMLGLLMKVAVPMCAPTDYTIYIERQECNYCVAVNTTICMGFCFSRDSNIKELVGPRFIVQRGC
*Danio*_TSHβrp XM_001341527.1	MRVLLCSF-LLLLGEDALLACSLKNYTLYVEKHECGHCMAINTTVCSGMCFTRDTNVQGFVGKRFLLQQSC

*Callorhinchus*_FSHβ	SYKEVIYQTITIPNCPSNVSPYYTYPVAISCQCGMCNTETTDCTVS--ALEPKYCSFTQQRKKRSLIMHSTLLHRNI*
*Callorhinchus*_TSHβ	TFHNIRYMMIRLPGCPPDIDPFYRLPVVLSCQCSQCATETTDCTNDIANQNPYHCTKPQWRIPATNSRIFIL*
*Callorhinchus*_TSHβ2	TFDELEYRTVRIPGCPTGVSSQHSYPTALSCKCKNCDTDYTDCTVQE-NLEANVCRKPQSETNSQD*
*Danio*_TSHβ	TYQEVEYRTAVLPGCPSHADPHFTYPVALSCHCSTCKTHSDECALRT-RSAGMRCSKPVHHLYPEENNYAQAYWDQYE*
*Danio*_TSHβrp	MHRSLVYRSARMPGCPVHIDPLFFYPVARRCNCTKCNTSRNECVFRH-KHKHNRCSKQLRTV*
