# Supplementary material for: Emergence and evolution of the glycoprotein hormone and neurotrophin gene families in vertebrates
Source: BMC Evol Biol. 2011 Nov 15;11:332. doi: 10.1186/1471-2148-11-332 (PMC3280201; doi:10.1186/1471-2148-11-332)
Supplement: Additional file 2 — Neurotrophin sequences. Amino-acid sequence of Callorhinchus milii (references from GeneBank), Scyliorhinus canicula, Petromyzon marinus and Lampetra fluviatilis (references from EMBL) NT encoding sequences characterized in this study aligned with Lampetra NT1 and human NT sequences for comparison (references from GeneBank). Cysteine residues involved in the cystine knot structure are in red. The amino acid sequence of the region of NTF4rp resulting from the shift in the open reading frame (see Additional file 3) is given in italic. [file 1471-2148-11-332-S2.PDF]

```
NTF4rp_Callorhinchus HQ174788      ----------------------------------------MIILLYAVVIS-----------LGRHRGWCCEPQRGSG
NTF4_Callorhinchus HQ174787        ----------------------------------------MIILLYAMAVSYFGGVETAPV-----------------
NTF4_Scyliorhinus FR693756         ----------------------------------------MITLLYTMVVLHLGSIKAAPP-----------------
NTF4_Homo NM_006179.1              ----------------------------------------MLPLPSCSLPILLLFLLPSVPIESQPLQPNST------
BDNF_Callorhinchus HQ174786        ----------------------------------------MTILFLTMVISYFGCLKAAPMKEAGGRGQGGSLYPALR
BDNF_Homo AF400438                 ----------------------------------------MTILFLTMVISYFGCMKAAPMKEANIRGQGGLAYPGVR
NTF3_Callorhinchus HQ174789        ----------------------------------------MSILFYGMLLTYLGGIQATPPTDRK---SLSENSVNSL
NTF3_Scyliorhinus FR693755         ---------------------------MVSSVTLLQVNKVMSVLFYGMLLTYLGGIQATTMDKRR----SSEDSVNSI
NTF3_Homo BC107075                 ----------------------------------------MSILFYVIFLAYLRGIQGNNMDQR----SLPEDSLNSL
NGF_Callorhinchus HQ174790         ------------------------------------MNEVMSILCCVLLITYLSATQASPGKEGYV------------
NGF_Homo NM_002506.2               ----------------------------------------MSMLFYTLITAFLIGIQAEPHSESNV------------
NT2_Petromyzon BN001521            ----------------------------------------MRFVLLTMLTMFIKGPAAAPIHEQHAV-VISDDSARTL
NT2_Lampetra FR693754              ------------------------------------------------------------------------------
NT1_Petromyzon BN00150             MRGLIEKGNDEEDDDREDDTEDGLDEVKEAQNKAMEEEEEEPVAVLDEVDGQKTTIKDLVDEAAVDKKAVSKEVDQEE
NT1_Lampetra AF071432              --------------?REHDTEDGYDEVKEVQNKAM--EEEEPVAVLDEVDGQKTTITDLVDEPAVDKKAVTKKVDQEE
NTz_Petromyzon BN001522            --------------------------------------------------------------------------????

NTF4rp_Cm      FPGERLPARQ---------------------------------------------PEGGEKEQDLYSLRVMWASEPPVNSPLPFIGEESQS---RADMG
NTF4_Cm        -------------NLSVAMPGPQAE------------QRAAGTNDSLGSDFQHVSPESWEKEWDLYSPRVMLASEPPGIPPLLFIMEASLS---QAEVA
NTF4_Sc        ---------------LLNHRSPQDELGRESRLNDEEAAGREYLLDDMVPDIQDLGDDASGREWDFYSPRVTLAARPPGVPPLLFIMEESLS---QADVA
NTF4_Hs        ---------------------------------------------------PSTLPPFLAPEWDLLSPRVVLSRGAPAGPPLLFLLEAGAFRESAGAPA
BDNF_Cm        PHGTLDNLISGGGGGGRGGGGAGGGGGGNGELGSLANTFEHVLEELLEVEQEPR-PSEGGKDVDLYSSRVMVSGQVPLEPPLLFLMEEYRN---YLEAA
BDNF_Hs        THGTLESVNGPKAGSR--------------GLTSLADTFEHVIEELLDEDQKVRPNEENNKDADLYTSRVMLSSQVPLEPPLLFLLEEYKN---YLDAA
NTF3_Cm        IIKIIQADILKAKLSKQTEGVKEKYQDTRYKSDALKNTESMGNTMSDFQPIISVNAELLRQKKRYHSPRVLLSERPPLEPPPLYLIDDFIG---DSRMG
NTF3_Sc        IIKILQADILKGRLAKQSEEVKEKYQDTKQKDDTHRNTNSLGNTISDFQPIVLVDAELLRQKKRYHSPRVLLSDRLPLQPPPLYLIEDFAE---SLEMD
NTF3_Hs        IIKLIQADILKNKLSKQMVDVKENYQSTLPKAEAPREPERGGPAKSAFQPVIAMDTELLRQQRRYNSPRVLLSDSTPLEPPPLYLMEDYVG---SPVVA
NGF_Cm         ------------TVG-VSFSPSLHNQSQSRVPQSGREMGHHSSNNVNGLHHFTVEPKLFKK-RRLYSSRVCFRNRSPPGLAGSLDQSRN-----DAQE-
NGF_Hs         --------PAGHTIPQVHWTKLQHSLDTALRRARSAPAAAIAARVAGQTRNITVDPRLFKK-RRLRSPRVLFSTQPPREAADTQDLDFEVG---GAAPF
NT2_Pm         LIPLLRTEMLKEVLLKTLQEEVGWGDSHPRRARDTGSQQQQQQPRPRISVIDAGLLSHVDEEELFSSRVILSELPPSGPPLLFPVDDFSA--SVVSVA
NT2_Lf         ---------------------------------------------------------------------????EQPPSGPPLLFPVDDFSA--SVVSVA
NT1_Pm         ANKVVDTNALLEQEGVMFDKGGKEAAVAPGAGGET-IRGASGKEAADRHYAAADHEEPAKNSGEPETEKVLGPFEREALSNAPSTRR-------ASGMS
NT1_Lf         ADKVVDTDALVEQEDVMFDKGGEEAAVAPGAGGETHHHGASGKEAADRPDAAADHEEPAKNSGELETEKVVGPFEREGLLSAPSHRR-------ASGRS
NTz_Pm         MAPRRPPMPCVLQHLKLRGASAATARPRWGADPEGNATRPRGRAPRGSDDATLAGAESPTR-SEPEPRAAGRLDGGDGDDGDEYDEEYEEEGDAEPFEA

NTF4rp_Cm      -DGTERARRKADNP-------------------------TFLGNLAICNSINSWVL--DKKTALDQYGETVTVLDMAP------------------SPS
NTF4_Cm        -NRTERARRQAGGE---------------------QVKPTRRGELSVCDSINFWVT--DKRTAVDINGWVVSVLNEVP------------------TSK
NTF4_Sc        -NRTWRAKRQAGAG---------------------GVDPSRRGDLSVCDSFSRWVT--DRRAAVDVHGKMVTVMIEVP------------------TST
NTF4_Hs        -NRSRRGVSETAPA-------------------------SRRGELAVCDAVSGWVT--DRRTAVDLRGREVEVLGEVPA-----------------AGG
BDNF_Cm        -NMSARVRRHSDP--------------------------ARRGELSVCDSISQWVTAEAKKTAVDMSGQTVTVLEKIL------------------VPN
BDNF_Hs        -NMSMRVRRHSDP--------------------------ARRGELSVCDSISEWVTAADKKTAVDMSGGTVTVLEKVP------------------VSK
NTF3_Cm        -NRTARRKRYADHK-------------------------GHRGEYSVCDSESRWVT--DKTAATDIRGRQVTVLGEIK------------------TGT
NTF3_Sc        -NRTARRKRYADRR-------------------------GHRGEYSVCDSESRWVT--DKTAAIDIRGKQVTVLGEIK------------------TGN
NTF3_Hs        -NRTSRRKRYAEHK-------------------------SHRGEYSVCDSESLWVT--DKSSAIDIRGHQVTVLGEIK------------------TGN
NGF_Cm         -NHGKRSRRVRRRVS----------------------GLRHRGLFSVCDSVHSWNQ--NKKSAIDIRGREVTLLSEFY------------------MNN
NGF_Hs         -NRTHRSKRSSSH------------------------PIFHRGEFSVCDSVSVWVG--DKTTATDIKGKEVMVLGEVN------------------INN
NT2_Pm         -NSTHHRRDRRHASSSSSSS-SSSSSSSSLSSSSGDPHRADRGELSVCDSESVWVT--DKSTAVDIKGNRVTVLDEMR------------------TST
NT2_Lf         -NSTHHRRDRRHASSSSSSS--SSSSSSSSSSSSGDPHRADRGELSVCDSESVWVT--DKSTAVDIKGNRVTVLEEMR------------------TST
NT1_Pm         KGNARRRSRSAAPQQQQKQQ-----------KQQQPPRRPHRGEYPACDSESRWVS--DRDTAFDITGMRVRVLGELSLSSSSASSSSSSSSSSSQSSS
NT1_Lf         KGKARRRSRSAAPQQQQKQQQQKQQQQQKQQKQQQPSRRPHRGEYPACDSESRWVS--DRDTAFDITGMRVRVLGELSLSSSSAS-SSSSSSSSSQPSS
NTz_Pm         TGHPRRRPRARRQAAERR--------------------PPHRAELAVCDSENVWVT--DKAHAVDLKGHNVTVLSDIR------------------VEG

NTF4rp_Cm      GPTKQIFFEVTCP-----------------------NTPTSR--CGGTDEKNRTFECKPRQSLVKAMTMDSKKKKGWRLIRVNTTCVCALKE*------
NTF4_Cm        GPMKQFFYETKCN----------------------NNTSTARSGCRGVDKRHWVSECKTKQSFVRALTVDHRKQAGWRWIRIDTACVCALNNRTTRT*-
NTF4_Sc        GPLKQYFYETKCN----------------------ERSTTAQGGCRGVDKRHWISECKTKQSYVRALTVDTQKRAGWRWIRIDTSCVCTLINRTGRI*-
NTF4_Hs        SPLRQYFFETRCK-----------------ADNAEEGGPAGGGGCRGVDRRHWVSECKAKQSYVRALTADAQGRVGWRWIRIDTACVCTLLSRTGRA*-
BDNF_Cm        GQLKQYFYETKCN-----------------------PKGFTNEGCRGIDKKHWNSQCKTSQSYVRALTMDSRKRIGWRFIRIDTSCVCTLTFKRGR*--
BDNF_Hs        GQLKQYFYETKCN-----------------------PMGYTKEGCRGIDKRHWNSQCRTTQSYVRALTMDSKKRIGWRFIRIDTSCVCTLTIKRGR*--
NTF3_Cm        TAIKQYFYETRCR-----------------------EAKPVKNGCRGIDDKHWNSQCKTCQTYVRALSTENKKYLGWRWIRIDTSCLCALSRKLGRS*-
NTF3_Sc        SAIKQYFYETRCR-----------------------EAKPVKNGCRGIDDKHWNSQCKTSQTYVRALSTENNKYVGWRWIRIDTSCLCALSRKLGKS*-
NTF3_Hs        SPVKQYFYETRCK-----------------------EARPVKNGCRGIDDKHWNSQCKTSQTYVRALTSENNKLVGWRWIRIDTSCVCALSRKIGRT*-
NGF_Cm         TGIRQIFYETRCR-----------------------SRKPSHGGCRGVDWRHWESHCDTTDSQVRALVLDNN-QVKWNFIRIKTACVCVLTKKTWQP*-
NGF_Hs         SVFKQYFFETKCR-----------------------DPNPVDSGCRGIDSKHWNSYCTTTHTFVKALTMDG-KQAAWRFIRIDTACVCVLSRKAVRRA*
NT2_Pm         VPLRQYFFETRCK-----------------------ATGNTRDGCRGVDKKHWNSTCRTTQSYVRALTMEGTRHVGWRWIRIDTSCACALSSKSGRAY*
NT2_Lf         VPLRQYFFETRCK-----------------------ATGNTRDGCRGVDKKHWNSTCRTTQSYVRALTMEGTRHVGWRWIRIDTSCACALSSKSGRA*-
NT1_Pm         TSVKQYFYETRCKPAAAAGAARAAGSRTREAPSRFASGTGTGAACRGADELRWRSQCKTTQSFVRALTEDARGRLAWRWIRLDTACVCTLTRRYGGA*-
NT1_Lf         TSVKQYFYETRCKPAAAAAGT---GIRSREAPSRFASGTGTGSACRGADELRWRSQCKTTQSFVRALTEDARGRLAWRWIRLDTACVCTLTRRYGGA*-
NTz_Pm         TALRQYFFETRCR-----------------------ASRHTRDGCRGIDRAHWSSQCRTVQSFVQALTSKGGRNLGWRWIRIDTACVCALSQRRP*---
```
