# Supplementary material for: Emergence and evolution of the glycoprotein hormone and neurotrophin gene families in vertebrates
Source: BMC Evol Biol. 2011 Nov 15;11:332. doi: 10.1186/1471-2148-11-332 (PMC3280201; doi:10.1186/1471-2148-11-332)
Supplement: Additional file 3 — Elephant shark NTF4 and NTF4rp. Comparison of NTF4 [Genebank:HQ174787] and NTF4rp [Genbank:HQ174788] sequences from Callorhinchus milii. Nucleotide deletions causing a shift in the open reading frame are indicated by red dashes and resulting amino acids given in italic. Other differences are highlighted in grey. [file 1471-2148-11-332-S3.PDF]

```
            M  I  I  L  L  Y  A  V  V  I  S     L  G  R  H  R  G  W  C  C  E  P  Q  R  G  S  G
NTF4rp     ATGATCATCCTCCTTTACGCCGTGGTTATTTCC--CTTGGGAGGCATCGAGGCTGGTGCTGCGAACCTCAACGTGGCAGTGGG----------
NTF4       ATGATCATCCTCCTTTACGCCATGGCTGTTTCCTATTTCGGAGGCGTCGAGACCGCTCCTGTGAACCTCAGCGTGGCGATGCCAGGCCCCAG
            M  I  I  L  L  Y  A  M  A  V  S  Y  F  G  G  V  E  T  A  P  V  N  L  S  V  A  M  P  G  P  Q

                                          F  P  G  E  R  L  P  A  R  Q  P  E  G  G  E  K  E  Q  D  L  Y
NTFrp      -----------------------------TTCCCTGGAGAGCGACTCCCAGCCCGTCAG-CCCGAGGGGGGGGAGAAGGAGCAGGATCTGTAC
NTF4       GCCGAGCAGCGGGCAGCGGGCACCAACGACTCCCTGGGGAGCGACTTCCAGCACGTCAGCCCGGAGTCGTGGGAGAAGGAGTGGGATCTGTAC
            A  E  Q  R  A  A  G  T  N  D  S  L  G  S  D  F  Q  H  V  S  P  E  S  W  E  K  E  W  D  L  Y

            S  L  R  V  M  W  A  S  E  P  P  V  N  S  P  L  P  F  I  G  E  E  S  Q  S  R  A  D  M  G  D
NTFrp      TCCTTGCGGGTCATGTGGGCTTCCGAGCCGCCTGTCAATTCTCCGCTGCCTTTCATCGGGGAAGAGTCGCAGAGCCGGGCTGACATGGGCGAC
NTF4       TCTCCGCGGGTCATGTTAGCCTCCGAGCCGCCCGGGATTCCTCCGCTGCTCTTCATCATGGAAGCTTCGCTGAGCCAGGCCGAGGTGGCCAAC
            S  P  R  V  M  L  A  S  E  P  P  G  I  P  P  L  L  F  I  M  E  A  S  L  S  Q  A  E  V  A  N

            G  T  E  R  A  R  R  K  A  D              N  P  T  F  L  G  N  L  A  I  C  N  S  I  N  S  W
NTFrp      GGGACGGAGCGGGCGAGACGCAAGGCCGAT------------AATCCCACCTTCCTCGGGAATCTCGCAATCTGCAACAGTATCAACTCCTGG
NTF4       AGGACAGAGCGGGCCAGGCGGCAGGCGGGGGGGGAGCAGGTCAAACCCACCCGCAGGGGCGAGCTCTCGGTCTGCGACAGCATCAACTTCTGG
            R  T  E  R  A  R  R  Q  A  G  G  E  Q  V  K  P  T  R  R  G  E  L  S  V  C  D  S  I  N  F  W

            V  L  D  K  K  T  A  L  D  Q  Y  G  E  T  V  T  V  L  D  M  A  P  S  P  S  G  P  T  K  Q  I
NTFrp      GTGTTGGACAAGAAGACGGCACTGGACCAATACGGAGAGACTGTGACTGTGCTGGACATGGCGCCCAGTCCCTCTGGACCCACCAAACAGATA
NTF4       GTGACGGACAAGCGGACGGCTGTGGACATCAACGGGTGGGTGGTGTCGGTGCTGAACGAGGTGCCCACCTCCAAGGGACCCATGAAACAGTTC
            V  T  D  K  R  T  A  V  D  I  N  G  W  V  V  S  V  L  N  E  V  P  T  S  K  G  P  M  K  Q  F

            F  F  E  V  T  C  P     N  T  P  T  S  R        C  G  G  T  D  E  K  N  R  T  F  E  C  K  P
NTFrp      TTCTTCGAGGTAACGTGCCCC---AACACGCCTACGAGCAGA------TGTGGGGAACAGACGAGAAGAATAGGACCTTCGAGTGCAAACCC
NTF4       TTCTACGAGACAAAGTGCAACAACAACACCAGCACAGCCAGAAGCGGCTGCCGAGGGGTAGACAAGAGGCACTGGGTCTCCGAGTGCAAAACC
            F  Y  E  T  K  C  N  N  N  T  S  T  A  R  S  G  C  R  G  V  D  K  R  H  W  V  S  E  C  K  T

            R  Q  S  L  V  K  A  M  T  M  D  S  K  K  K  K  G  W  R  L  I  R  V  N  T  T  C  V  C  A  L
NTFrp      AGGCAGTCCTTAGTCAAGGCGATGACTATGGATAGCAAGAAAAAAAAGGGGTGGAGGTTGATACGGGTAAATACCACTTGTGTCTGTGCCCTG
NTF4       AAACAGTCCTTTGTCCGGGCGCTGACTGTGGACCACAGGAAACAAGCGGGGTGGCGGTGGATAAGGATAGACACCGCGTGTGTCTGTGCCCTG
            K  Q  S  F  V  R  A  L  T  V  D  H  R  K  Q  A  G  W  R  W  I  R  I  D  T  A  C  V  C  A  L

            K  E  *
NTFrp      AAGGAGTAG
NTF4       AACAACAGAACTACAAGGACGTGA
            N  N  R  T  T  R  T  *
```
