# Supplementary material for: Emergence and evolution of the glycoprotein hormone and neurotrophin gene families in vertebrates
Source: BMC Evol Biol. 2011 Nov 15;11:332. doi: 10.1186/1471-2148-11-332 (PMC3280201; doi:10.1186/1471-2148-11-332)
Supplement: Additional file 4 — Lin7 sequences. Alignment of Lin7-related encoding sequences of Scyliorhinus canicula, Callorhinchus milii (Callo) and Petromyzon marinus characterized in this study with the amphioxus Branchiostoma floridae homologue and sequences from human (Homo), chicken (Gallus) and zebrafish (Danio). Three paralogues are evidenced in Scyliorhinus and two in Petromyzon. References are from GeneBank except for Scyliorhinus sequences that are from EMBL. Positions specific to Lin7b sequences are highlighted in blue, those specific to Lin7a are in red and those to Lin7c are in green. Amino acids identical to Branchiostoma are given by dashes. Gaps are identified by dots. Unknown positions are indicated by interrogation marks. Exon boundaries are indicated in the bottom lines. [file 1471-2148-11-332-S4.PDF]

```
Branchiostoma (XM_002246298)  MAST..GEALTLERDVRRAIELLDNLQKTGEVPPQKLQALQKVLQSDFCNAIREVYEHVYETVDLTGSPEIRANATAK
1_Petromyzon (BN001523)       ???????????????IA------EK--RS----SE------R----E--T------E--Y---GIS--A-V--S----
2_Petromyzon (BN001524)       --TL-AV-P-R-D---A------ER--RS----AH-IL---K-------T-V----EYIY--L-I--NQ-M--L----
B_Homo (NP_071448.1)          --AL..V-P-G-----S--V---ER--RS--L---------R----R--S-------QL-D-L-I---A----H----
B_Gallus (CN234764.1)         --ALS.ADP-G-----A--V---ER--RS--M---------R----K--C-------QL-D-L-IA-------H----
B_Danio (NP_001018569.1)      --AM..T-P-C-----C-V-----R--RS-QL--P------R----K--A-------QL-D-L-II-G--V--Q----
B_Scyliorhinus (FR693758)     --AL..ADS-S-----A------EK--RS-N-------T--R----K--S-------QL---L-IN-------H----
A_Homo (NP_004655.1)          --TLTVVQP---D---A------EK--ES----VH---S-K-----E--T------QYMH--ITVN-C--F--R----
A_Gallus (XP_416117.2)        --TLTAVQP---D---A------EK--ES----VH---S-K-----E--T------QYMH--ITVN-C--F--R----
A_Danio (NP_957003.1)         --TVT.VQP-------T------EK--ES-D--GH---S-K-----E--T------QYMH--ITVN-C--YQ-R----
A_Callo_3 (AAVX01351753.1)    ......................................................--QYMH--ITIN-C--F--H----
C_Homo (NP_060832.1)          --AL----PVR----IC------EK--RS------------R----E----V----E--Y----ISS---V-------
C_Gallus (NP_001026238.1)     --AL----PVR----IC------EK--RS------------R----E----V----E--Y----ISS---V-------
C_Danio (NP_001004672.1)      ---L----PVR----IS-------K--R-------------R----E--K-V----E--Y----INS---V-------
C_Scyliorhinus (FR693759)     ---LV-M-PIR-D---S------EK--RS------------R----E--I-V----E--Y----INS---V-------
C_Callo (HQ174794)            ???????????????-S------EK--RS--------H---R----E--I-V----E--Y----INS---V--S----
                              <--- exon 1 --><--------------- exon 2 -------------><------- exon 3 -------->

Branchiostoma                 ATVAAFAASEGHAHPRVVELPKTEEGLGFNVMGGKEQNSPIYISRVIPSGVADRHGGLKRGDQLLSVNGV
1_Petromyzon                  ------------------------------I--------------I--G-----Q---------------
2_Petromyzon                  ---------D-----------------------------------I--G-----Q---------------
B_Homo                        ------T-----------------------I-----------------G---------------------
B_Gallus                      ------------------------------I-----------------G---------------------
B_Danio                       ------------------------------I-----------------G-----Q---------P-----
B_Scyliorhinus                -----------------------E------I--------------I--G---------------------
?_Callo_4/2 (AAVX01085532.1)  ------------S----I-----E---------------------I--G---------------------
A_Homo                        ------------S--------------------------------I--G---E-----------------
A_Gallus                      ------------S--------------------------------I--G---E-----------------
A_Danio                       ------------S--------------------------------I--G---E-----------------
A_Sciliorhinus (FFR693757)    ...---------S----------E---------------------I--G---------------------
?_Callo_4/1 (AAVX01548863.1)  ------------S-----------------I--------------I--G-I-------------------
C_Homo (FSH)                  ------------S----------E------I--------------I--G-I-------------------
C_Gallus                      ------------S----------E------I--------------I--G-I-------------------
C_Danio                       ------------S----------E------I--------------I--G-I-------------------
C_Scyliorhinus                ------------S-----------------I--------------I--A-I-------------------
C_Callo                       -----------------------E------I--------------I--G---------------------
                              <------------------------------- exon 4 -----I----------------------->

Branchiostoma                 SVEGENHEKAVELLKAATGTVKLVVRYTPKVLEEMEARFEKMRNNRRGRRPMEC*.................
1_Petromyzon                  -----H---------Q-QG----------K------A----L-TA--RQTHNNSFS*...............
2_Petromyzon                  -----H---------Q-Q-S----------------A------TAK-RQQSTSS*.................
B_Homo                        -----Q-----------Q-S---------R------A------SA--RQQHQSYSSLESRG*..........
B_Gallus                      -----Q---------?-QSC-RF------R------A----L-SA--RQ-?YSYSS................
B_Danio                       -----H-----------QGS----------------A------SA--RQQHTSYSSLESRG*..........
B_Scyliorhinus                -----Q-----------Q------------------A------TA--RQQHNSYSSLESRG*..........
A_Homo                        -----H-----------KDS----------------A----L-TA--RQQQQLLIQQQQQQQQQQTQQNHMS
A_Gallus                      -----H-----------KDS----------------A----L-TA--RQQQQLLIQQQQQQQN..TQQNHMS
A_Danio                       -----H-----------KDS----------------A----L-TA--RQQQQILMQQQQQQNLA.AQQNHMS
A_Scyliorhinus                -----H-----------QSS----------------A----L-TA--RQQQQILLQQQQQQQQ..VQQNHMS
C_Homo                        -----H-----------Q-N----------------S------SAK-RQQT*....................
C_Gallus                      -----H-----------Q-K----------------S------SAK-RQQN*....................
C_Danio                       -----H-----------Q------------------S----L-SAK-RQQNNFPK*................
C_Scyliorhynus                -----H-----------H------------------S------SAK-RQQINY*..................
C_Callo                       -----H-----------H------------------S------SAK-RQQINY*..................
                              <------------------------------- exon 5 ------------------------------->
```
