# Supplementary material for: Emergence and evolution of the glycoprotein hormone and neurotrophin gene families in vertebrates
Source: BMC Evol Biol. 2011 Nov 15;11:332. doi: 10.1186/1471-2148-11-332 (PMC3280201; doi:10.1186/1471-2148-11-332)
Supplement: Additional file 5 — Elephant shark KCNA sequences. Callorhinchus milii KCNA sequences. Complete sequences were deposited at GenBank and are given with their accession number. Other, partial sequences are given with their reference from the genome survey sequence at GenBank. Sequences were numbered according to their phylogenetic relationship with other members of the vertebrate KCNA family (see Figure 3). [file 1471-2148-11-332-S5.PDF]

KCNA_1 [GENBANK:HQ174791]

ATGACTGTTTTAGCAGGCGACAACATAGATGAAGCCTCCGCCTTGCCTGGACACCCTCAAGACAGTTACCAACCC
GACCATGAGGACCACGAGTGTTGTGAGCGGGTGGTCATCAACATCTCGGGGCTGCGGTTCGAGACCCAGCTCAAG
ACCCTGTCCCAGTTCCCCAACACCATGCTGGGCAACCCCAAGAAGAGAATGCGCTATTTCGACCCCTTGCGAAAC
GAGTACTTCTTCGACCGCAACCGCCCCAGCTTCGACGCCATCCTCTACTATTACCAGTCAGGTGGCCGGCTAAGG
AGACCCGTCAACGTGCCCCTGGACATGTTCTCGGAGGAGATCAAATTTTACGAGCTGGGGGAAGAGGCAATGGAG
AAGTTCCGCGAGGATGAAGGCTTTATCAAAGAGGAGGAACGCCCATTGCCAGAGAATGAGTACCAACGTCAAGTC
TGGCTCTTATTCGAGCATCCCGAGAGTTCTGGACCAGCCAGGGGCATTGCCATAGTCTCGGTCATGGTCATTTTG
ATCTCCATCGTTATCTTTTGCTTGGAAACCCTGCCAGAGTTGAAGGATGACAAGGATGTTGCGAGTAGGACAGTG
ACCATTGGCAACGTCACGACTATTTACAAGCAGAACATCCTCACCGATCCCTTCTTCATCGTGGAGACCCTCTGC
ATCATCTGGTTCTCCTTTGAGCTGATCGTCAGGTTTTTCGCCTGTCCCAGCAAAGCGGAGTTCTTCAAGAACATC
ATGAACTTCATTGACATCGTGGCCATCATCCCCTATTTCATCACCTTGGGCACCGAACTGGCCGATGAAAAAGGG
GAGGAGAATAAGAGTGGGGAGCAAGCCACCTCTTTGGCCATCCTAAGGGTCATTCGGCTGGTTCGGGTGTTCAGG
ATTTTCAAGCTGTCCAGGCACTCCAAGGGCCTGCAGATTCTAGGGCAGACTCTCAAAGCCAGCATGAGAGAGTTG
GGACTGCTTATATTTTTCCTCTTCATTGGAGTCATCTTGTTCTCCAGCGCCGTGTTCTTCGCTGAAGCTGACGAC
CCCGATTCCTTGTTCACCAGCATCCCAGATGCTTTCTGGTGGGCTGTCGTGTCCATGACCACTGTGGGCTATGGC
GACATGTACCCCGTCACCATCGGGGGTAAAATCGTGGGGTCACTGTGTGCCATCGCTGGGGTGTTGACCATCGCC
CTCCCTGTGCCTGTCATCGTCTCTAACTTTAACTACTTCTACCATAGAGAGACCGAAGGGGAGGAACAGGCTCAG
TACCTGCACGTTAGTTGCCCCAACATCGCTTCAGAGAGCGACGTGAGAAGCCACAGCTCCACCACCATCAGCAAA
TCCGAGTACATGGAGATTGCGGAAGACATGAAAAACAGCATAGACAACTTCAGAGAAGCCAATCTTAGAACTGGC
GAGTGGACTGTAGCAAACCAAAACTGTGTGAATAAAAACAAGCTGCTGACAGATGTTtaa

KCNA_2 [GENBANK:HQ174792]

ATGACCGTAGCCGCAGGGGACAGCGGAGACGAGGTGGTGGCCGCTCCACCAACCTCCGAGCTGGAGCACGAGTGC
TGCGAGCGGGTGGTGGTCAACATCTCGGGGCTGCGCTTTGAGACGCAGCTCAAGACGCTGTCGCTCTTCCCGGAC
ACGCTGCTGGGAGACCCCAGGAAGCGGATGCGGTATTTCGACCCCCTGAGGAATGAGTATTTCTTCGACAGGAAC
CGCCCGAGCTTCGACGCCATCCTCTACTACTACCAGTCCGGGGGCAGGATCCGCAGACCCGTCAACGTGCCCATC
GACATCTTCTCTGAGGAACTCCGCTTCTACCAGCTGGGCGAGGAGGCCATGGAGATGTTCAGGGAGGACGAGGGC
TTCATCCAGGAGAAGGAGCGCCCTTTGCCGGCCAACGAGTTCCAGCGCCAGGTGTGGCTTCTCTTCGAGTATCCC
GAGAGCTCGGGTCCAGCCCGGGCCATCGCGATCGTGTCTGTGATGGTCATCCTCATCTCCATCGTCAGCTTCTGC
CTGGAGACCTTGCCCATGTTTCGAGAAGAGGCAGAGCACCTCGGCGCCCCCCGACCCCAACACACCAACAGCACA
GAGCCTTATCAATCCGGCACCTTCACTGACCCCTTCTTCATCCTCGAGACCCTCTGCATCATCTGGTTCTCCTTT
GAGTTTCTGGTCCGATTGTTCGCCTGCCCCAGCAAGCCCATCTTCTTCAAGAACATGATGAACATCATCGACATC
GTGGCCATCATCCCTTACTTCATCACCCTGGGCACCGAGCTGGCAGCTCAACAGCAACAGCATCAGGAGAAGGAG
GAGGGAGCAGGGCAACAACAAGGGCAGCAAGCCATGTCTCTGGCCATCCTCAGGGTTATCCGCCTGGTGCGAGTC
TTCAGGATTTTCAAGCTCTCCAGACACTCCAAGGGGCTGCAGATCCTGGGGCAGACCCTGAAGGCCAGCATGAGG
GAGCTGGGGCTCCTGATCTTCTTCCTCTTCATCGGAGTCATCCTCTTCTCCAGCGCCGTCTACTTCGCCGAGGCT
GATGAGAAAATGTCCTACTTCAACAACATCCCCGAAGCCTTCTGGTGGGCGGTGGTGACCATGACCACCGTAGGC
TATGGGGACATGTACCCCATCACCATCGGGGGCAAGATTGTGGGGTCGCTGTGCGCCATCGCCGGGGTCTTAACC
ATCGCCCTGCCCGTTCCCGTCATCGTCTCCAACTTTAACTATTTCTACCATCGGGAGACGGAGGGTGAGGAGCAA
GCTCGGTATCTACACATCACCAGTTGTCCCCGGCTAAAGGCCAGCAGCGAGCTGCCGAGGAGTCCCATCGCCTCT
AGCTTGGACAATGATGACTATATGGATCCCCGAGAAGCGGGCGACCCTCCCGAGGAGGACAGACCAGAGACTCCA
CATTGCCTTTTACCCAACACCAACCATGTCAACATCACCAAAATGCTGACTGACGTTtga

KCNA_3 [GENBANK:HQ174793]

ATGCAGCTGGTGGCGGGGGACGGCACGGAGCCGGCGGGGTCGCTGGTCGGCTGCCCGGAGCCCGAGCCCGAGCCC
GAGCCGCCGGAGCCCGAGCCCGAGTGCTGCGAGCGGGTGGTGGTCAACATATCGGGGCTGCGCTTCGAGACGCGG
CTCAAGACGCTGTCGCTCTTCCCGGACACACTGCTCGGAGACCCCAAGAAGCGGATGCGCTACTTCGACCCCCTG
AGGAACGAGTACTTCTTCGACAGGAACCGACCCAGCTTCGACGCCATCCTCTACTACTACCAGTCCGGGGGCAGG
ATCCGCAGACCCGTCAACGTGCCCATCGACATCTTATCCGAGGAGATCCGCTTATACCAGCTGGGCGAGGAGGCC
ATGGAGATGTTCAGGGAGGACGAGGGCTTCATCCAGGAGAAGGAGCGCCCTTTGCCGGCCCACGAGTTCCAGCGC
CAGGTGTGGCTTCTCTTCGAGTACCCCGAGAGCTCGGGTCCAGCCCGGGCCATCGCGATCGTGTCCGTCATGGTC
ATCCTCATCTCCATCGTCATCTTCTGCCTGGAGACCCTGCCCGAGTTCCGAGACGACAAGGACTACCGGCCCGGC
CCCGGCAGCCCGGTCTCGGCGCCCCAGGTCCGGGCCGACGCCAACGCCAACGCCACCGGCGACCCCCCCGACCCG
GCGTGGGGCGGCATCTTCGCCGACCCTTTCTTCGTGGTCGAGACCCTCTGCATCATCTGGTTCTCCTTCGAGCTC
CTGGTGCGCTTCTTCACCTGCCCCAGCAAAGCCAGCTTCGCCAAGAACATCATGAACATCATCGACATCGTGGCC
ATCATCCCTTACTTCATCACCCTGGGCACCGAGCTGGCCGAGAGGCAGGGCAACGGTCAGCAAGCCATGTCTCTG
GCCATCCTCAGGGTCATCCGCCTGGTGCGAGTCTTCAGGATCTTCAAGCTCTCCAGACACTCCAAGGGGCTGCAG
ATCCTGGGGCAGACCCTGAAGGCCAGCATGAGGGAGCTGGGGCTCCTGATCTTCTTCCTCTTCATCGGGGTCATC
CTCTTCTCCAGCGCCGTCTACTTCGCCGAGGCCGACGACCCCGGCTCGGGCTTCAGCAGCATCCCCGACGCCTTC
TGGTGGGCGGTGGTGACCATGACCACCGTGGGCTACGGGGACATGCACCCCATCACCATCGGGGGCAAGATCGTG
GGGTCGCTGTGCGCCATCGCCGGGGTCTTAACCATCGCCCTGCCCGTCCCGGTCATCGTCTCCAACTTTAACTAT
TTCTACCACCGGGAGACGGAGGGCGAGGAGCAAGCTCAGTATCTCCATGTCACCAGTTGTCCCCACCTGTCCCCT

TCCCCTTCCTCCTCCTCCTCCTCCTCCTGCACCGAGGAGCTGAAGAAGTCCCGCAGCAGCTCGTCCCTCAGCAAG
TCTGAGTACATGGTGATCGAGGAAGGCATCAACCACCACCACCACCACCACCACAAACAGGTCAACTTCCAAACC
GGGAACTGCACCGCGAACGCCCCGAACTGTGTGGCCATCAAGAAGATCTTCACCGACGTGtaa

KCNA_4 partial sequence: [GenBank:AAVX01360765.1] (complement 1-441)
???GGTCGGGGTCGGTGTGCAGCGCGTTCGAGTGCCGCGAGCGGGTGGTCATCAACGTGTCGGGCCTGAGGTTCG
AGACCCAGATGCAGACCCTCAGCCGCTTCCCGGACACGCTGCTCGGGGACCCGGACAAGCGGCTGCGCTACTTCG
ACCCCCTGCGGAACGAGTACTTCTTCGACCGGAACCGTCCGAGCTTCGACGCCATCCTGTACTACTACCAGTCGC
GGGGTCGGCTGAAGCGGCCGGTCAACGTGCCGCTGGACATCTTCTCGGAGGAGGTCAAGTTCTACGAGCTGGGGG
GCGAGGCCGTGCTCAAGTTCCGCGAGGAGGAGGGCTTCGCGCGGGACGCGGGGGGCGGTGGGGAGAGGGCGCTGC
CCGCCGGGGAGTTCCAGCGCCAGGTCTGGCTGCTGTTCGAGTACCCCGAGAGCTCGAGCCCGGCCCGGG???

KCNA_5 partial sequence: [GenBank:AAVX01076124.1] (113-1427)
ATGAACGACATCAACGTGGCCGGGGAGAACTCGCTGGATTCCATGGCAAGGGGGCGGCTGGAGCAGGACCAGGAC
CAGGTGCCTTCCGAGGACAACCGCTTGGAGTTGGAGTTCTTGGCCAGGAACAGGTCGGAGCGGGTGATCGTCAAC
ATCGCCGGCCTCAAGTACGAGACCCAACTGGGTACCATCAACCAGTTCCCGGACACCTTACTGGGCGACCCCCAG
AAAAGGATGAGATACTTCGACCCCTTGAGGAACGAGTACTTCTTCGACCGGAACCGCCCGAGCTTCGATGGCATC
CTGTACTTCTATCAATCCGGCGGGAAGATCAGGAGACCGGTCAACGTCTCCATCGACATCTTCGCCGACGAGATC
CGCTTCTACCAACTGGGCGCCGAGGCCATGGAGAGGTTCCGGGAGGAGGAGGGCTTCATCAAGGAGGAGGAGAAG
CCTCTGCCCAAGCAGGAGTTCCAACGCCAGCTTTGGCTGCTCTTCGAGTACCCCGAGAGCTCCAGCCCGGCCCGA
GGCATCGCCATCGTGTCCGTCCTGGTCATCGTCATCTCCATCATCATCTTCTGCCTGGAAACGCTGCCCGAGTTT
CGGGACGAGAGGTACAACCTCGTGGGCCCCAATGGCACCCACATCTCCTCCATCCACATGAGCCTGACCGACCCC
TTCTTCGTCATCGAGACCACCTGCGTGGTCTGGTTCACCCTCGAGCTCCTGATGAGGTTCTTCGCCTGCCCCAGC
AAGTCGGTGTTCTCCAGGGACATCATGAACATCATCGACGTGGTGGCCATCTTCCCCTACTTCATCACCCTGGGC
ACGGAGCTGGCGGAACAGCAGTCAAACGGCCAACAGGCCATGTCCCTGGCCATCCTGAGGGTCATCCGGCTGGTC
AGGGTCTTCAGGATCTTCAAGCTGTCCAGGCACTCCAAGGGGCTGCAGATCCTGGGCCAGACCCTGAAGGCCAGC
ATGAGGGAGCTGGGCTTGCTCATCTTCTTCCTCTTCATCGGGGTCATCCTCTTCTCCAGCGCCGTGTACTTCGCC
GAGGCGGATGAGCCGTCGTCCTTCTTCTCCAGCATCCCCGATGCTTTCTGGTGGGCGGTGGTGACCATGACCACC
GTGGGCTACGGGGACATGAGGCCGGTCACCATGGGGGGCAAGATCGTGGGCTCGCTCTGCGCCATCGCCGGGGTG
TTGACCATCGCCCTCCCGGTGCCGGTCATCGTGTCCAACTTCAACTATTTCTACCACCGGGAGACGGACAACGCG
GACCAGGGCAGCCTGAACGACGACGACAGCGCGGAGGGCA???
END

KCNA_6 partial sequence: [GenBank:AAVX01082292.1] (10-1289)
???GGGGACCCGGGCAAGAGGATGCGCTACTTCGACCCGTTGCGCAACGAGTACTTCTTCGACCGCAACCGCCCG
AGTTTCGACGCCATCCTCTACTTCTACCAGTCCCGGGGGCGGCTCCGGAGACCCGCCAACGTGCCCTTTGACGTC
TTCATGGAGGAGATCCGCTTCTACCAGTTGGGCGACCAAGCCATCGCCAATTTCCGAGAGGAGGAAGGCTTCTTC
CAGGAGGAGGAACGCGCCCTGCCGGAGGGCGAGTACCAGCGCCAGGTCTGGCTCCTCTTCGAGTACCCTGAGAGC
TCGGGTCCGGCCAAGGGCATCGCCATCGTGTCCGTGCTGGTCATCTTGATATCCATCGTTATCTTCTGCCTGGAG
ACCCTCCCCGAGTTCAGGGAGGACCAGACCCGGCTGCACATGTCCGAAACAGCGGCGAACGGTACCCAACTCGTG
GCCAGAGACAACCCCTTCACCGACCCCTTCTTCATCACTGAGACCCTCTGCATCATGTGGTTCTCCTTCGAGCTC
CTGGTCCGGTTCTTCTCGAGCCCCAGCAAGCCCGGCTTCTTCAAGAACATCATGAACATCATCGACATCGTGGCC
ATCATCCCCTACTTCATCACCCTCTTCACCGAGCTGGTGCACCATCAAAGCAACGGCCAACAGGCCATGTCCCTG
GCCATCCTCAGGGTCATCCGGCTGGTCAGGGTCTTCAGGATCTTCAAGCTGTCCAGGCACTCCAAGGGGCTGCAG
ATCCTGGGCAAGACTCTCCAGGCCAGCCTGAGGGAGCTGGGCTTGCTCATGTTCTTCCTCTTCATCGGGGTCATC
CTCTTCTCCAGCGCCGTGTACTTCGCCGAGTCTGACGACCCCGATTCCTCGTTCAACAGCATCCCCGAGGCTTTC
TGGTGGGCTGTGGTGACCATGACCACCGTGGGCTACGGGGACATGTACCCGGTCACCATCGGGGGGAAGATCGTG
GGCTCGCTGTGCGCCATTGCCGGGGTGTTGACCATCGCCCTCCCGGTGCCCGTCATCGTGTCCAACTTCAACTAC
TTCTACCACCGGGGGAGCGAGAACGACGAGCAGCCCCCTTACCCGCAGGGGAGCAGTGCGGGAGAGGGGTCAGGT
TCCTTGGGGGGCGATCTGAGCAGCAACCCATCGCTCCACAAATGCGAGTATCCAGAAGCCGAGTCCGACCTGATA
TGGCGAGAGGAGAGAAACGCCAATATCACGACTTCTCCCCCATCAAACAGAGCTTCGCAGACACCCAGAACATGC
TCACTCCT

KCNA_7 partial sequence: join [GenBank:ti|1573106156] (complement 616-527) [GenBank:AAVX01597379.1] (24-531)
ATGATTATTATTATGGCTCTTTTACCAGACGTTACCAGTTCACTCCACCACACAGGTAAGTCCCATAAACCCCCC
TCTCCCTACGAGCTTTGCAGCCAAGGGAATCATGAAGGATGTGAGCGAGTAGTCATCAATGTGTCGGGACTGCGT
TTCGAGACCCAACTCAAGACTCTCGGCCAGTTCCCGGACAGCTTGTTGGGAGATCCTCACAGGAGAATCCGCTAC
TTCGACCCCCTGAGGAACGAGTACTTCTTCGACAGGAACCGCCCCAGCTTCGACGCCATCCTCTACTACTACCAG
AGCGGGGGCCGGCTGAAGAGGCCGTCCACCATCCCCCTCGACGTCTTCATGGAAGAGCTGAGGTTCTACGAGTTG
GGCGACGACACCATCACCAGGTTTCGCGAAGACGAGGGCTTCACCAAAGAGGAGGAACGGCCCCTGCCAGACAAT
GAGTTCCAGAGGCAGATTTGGCTGCTGTTTGAGTACCCGGAGAGTTCAGCCCCGGCCAGGATCGTGGCTCTCATC
TCGGTCCTTGTCATCCTCATCTCCATCGTCATCTTCTGCCTGGAGACACTGCCCGAGTTAAGGAGGAGAAGGG

KCNA_10 partial sequence: [GenBank:AAVX01075604.1] (complement 1816-705)

```
???GCCATTGGACGTCTTTGCGGAAGAGATCCACTTCTACGAGCTGGGCGCGGCGGTGATGGAACAGTTCCGGGA
GGGCGAGGGTTTCATCAAAGAGGAAGAGGTCCAGTTACCGGTCAACCAGACCCACAAGCAGTTCTGGCTCCTCTT
TGAGTACCCGGAGAGTTCTGGTGCTGCCAGAGGTGCTGCGCTGGTCTCCGTCTCCATCATTGTGGTGTCTATCGT
CAACTTCTGCCTGGAGACCCTCCCTGAATTCCAGGACAACCGTGAGTTGAGAGATTCCACCCAGCCTGCTAACTT
GAGTCAGGCCCTGGAGTCCAGCTCCCAACATAACATCTTCAGTGACCCTTTCTTCCTGGTGGAGACCGCCTGCAT
CTTCTGGTTCTTTGCAGAACTGTGTATCAGGTTCGCTGCTTGCCCCAGCAAGCCCGAGTTCTTCAAGGCAATCAT
GAACATCATTGACCTGATGTCCATCATCCCATACTTTGTCACTCTCATCACCGAACTGGTACAGGAACAACAAGA
AGCCGATGGACAGCAGCAGACCACGTCACTTACCATCCTGAGGATCATCCGCCTTGTGCGAGTTTTCAGGATCTT
CAAGCTCTCCAGACACTCCAAGGGGCTGCAGATCCTGGGGAAAACCCTGAAAGCCAGCCTGAAGGAGCTGGGGCT
CCTTATCTTCTTCCTCTTCATCGGGGTCATCCTCTTCTCTAGCGCCGTCTACTTTGCCGAGGTGGATGAGCCGAA
CTCGCAGTTCTCCAGCATCCCCGATGGCTTCTGGTGGGCGGTGGTGACCATGACCACAGTGGGCTATGGGGACAT
GTGCCCCATCACTCTGGGGGGCAAGTTGGTGGGCATGCTGTGCGCCATCGCTGGGGTGTTAACCATCGCCCTGCC
CGTTCCTGTCATAGTCTCCAACTTTAACTACTTCTACCACCGGGATAGAGAGAATATGGAGAAGCAATGCATCAC
CATTACTGATGAGAATGCCCCCCCACTGAGCGAGGGCTCAGTCACCCGTGACTGCATCGTCTCCATTAACAAGGT
TGACGGAGTCTATGTGGGCGGAGAGGAGGGGGCAGCCATGTGGAAGGAATGGCTCTGTGGCAGACGAACCTTCAA
CATTCGCACTTTCCTCTCCCGCAAGACCTCCtga
```
