# Supplementary material for: Emergence and evolution of the glycoprotein hormone and neurotrophin gene families in vertebrates
Source: BMC Evol Biol. 2011 Nov 15;11:332. doi: 10.1186/1471-2148-11-332 (PMC3280201; doi:10.1186/1471-2148-11-332)
Supplement: Additional file 6 — Lamprey KCNA sequences. Petromyzon marinus KCNA-related sequences extracted from their Contig sequences on the Washington University dedicated server (see Methods) and/or from the Traces archives deposited in GeneBank. The positions in the Contig or trace sequences in the sense or antisense strand (complement) are indicated. The underlined position corresponds to the first nucleotide of the 5' end ATG when appropriate. KCNA sequences were referenced by letters instead of numbers because their relationship with the different known KCNA-types could not be determined. [file 1471-2148-11-332-S6.PDF]

KCNA_A Contig6970.3 (complement 7274-5841)
ATGGACGACGCGGCGTTGCTTGAAGATGGCCCCGAGACGGCAGAGCCGCTGACACACGAGTACTGCGAGAGGATC
GTCATCAACGTGTCGGGACTGCGCTTCGAGACGCAGCTCAAGTCGCTCGACCAATTCCCGGACACCCTCCTGGGC
AACCCCAGCAAGAGGTCGCGCTACTTCGACCCACTGCGCAACGAGTACTTCTTCGACAGAAACCGGCCAAGCTTC
GACGCCATCCTCTACTACTACCAGTCCGGCGGGCGGCTAAGGCGCCCGGTCAACGTCTCCATCGACGTGTTCAGC
GAGGAGATCAAGTTCTACGAGCTCGGCAACGAGGCCATGGCCAAGTTCCAGGAAGACGAGGGCTTCATCAAGGAG
GAGGAGAAGCCGCTGCCGTCGGACGAGTTCCAGCGGCAGGTGTGGCTGCTGTTCGAATACCCCGAGAGCTCCAAC
CCGGCCAGGGGAATTGCCATCGTTTCCGTACTGGTCATTCTCATCTCCATCATCATATTCTGCCTCGAGACTCTG
CCTGAATTCAGGGACGAGAAGGAACAGAAATTTCTCCCAGCAAAGGACATCAATGGCACACAGGTAAAAATTATG
CATGGCAGCTCTTTCACTGATCCATTTTTTATTGTGGAAACCATTTGCATCATTTGGTTTTCCTTTGAACTGCTG
GTGAGGTTTTTTGCGTGCCCCAGCAAACCAGGATTTTTCAAAAGCATCATGAACATCATTGACATCGTGGCCATT
CTGCCCTACTTCATTACCCTTGGCACCGAGATGGCCAAGCACCAAGGGAGTGGCCAGCAGGCCATGTCACTGGCG
ATTTTGAGGGTGATCCGGCTGGTGCGAGTGTTCCGGATATTCAAGCTGTCCAGGCACTCCAAGGGCTTGCAGATT
CTGGGCCAGACTTTGAAGGCGAGCATGAGAGAGCTGGGCCTTCTCATATTTTTCCTCTTCATCGGCGTCATCCTC
TTCTCCAGCGCCGTATACTTTGCCGAGGCCGACCACCAAAACTCGGAGTTCCGCAGCATCCCCGACGCCTTCTGG
TGGGCCGTCGTAACCATGACGACGGTGGGCTACGGCGACATGCGTCCCGTGACGGTGGGTGGCAAGATCGTGGGC
TCGCTGTGCGCCATCGCCGGCGTGCTCACCATCGCCCTCCCCGTGCCCGTCATCGTCTCCAACTTCAACTACTTT
TACCACCGCGAGACGGACAACGAAGAGCACGCGAACTACCTGCACGTCTCAAGCTGCTCGCACCAGGAGGAGAGC
AGAAAGAGCAGGAGCCCGTCCATGAGCAGGTCCGAATACATGGAGATGGACGACGGTCTGAGCAACGACGTTGAC
TTCAAGGTGAAGATGCCCCTCAAGCCCAACAACTGCGCGCCGCTCAACCACAACTGCATCAACAGAAACGTCGAG
ACCGATGTCtga

KCNA_B Join Contig12452.3 (2948-3415) Contig12452.4 (4-1006)
ATGACCATCGCCAGCGCCAACAACAGCATCACGGGCCATGGCAGCACGGGCAGCCTCTACAATCCGACGAACGAG
ACGCGTGACTGCATGCGTGCCGTCACGCGAGAGGACATTCACGCGCACCTCCTCGACGGCAACGAGCGCGTGGTC
ATCAACGTGGCCGGCATGAAGTTCGAGACGTGCGCGCGGACGCTCGCCCAGTTCCCCGACACGCTGCTGGGCGAC
CCCGCGCGACGCGCCCAGCACTTCGACCCCCTGCGCAACGAGTACTTCTTCGACCGCAACCGGCCCAGCTTCGAC
GCCATCCTCTACTACTACCAGTCGGGCGGGCGGCTGCGCAGGCCCGCCAACATCCCCTTCGACCTGTTCTCCGAG
GAGATCCGCTTCTTCGAGCTGGGCGAGGAGACGATCCAGCGCTTCCGCGAGGATGAGGGCTACATCCGCGAGGAG
GAGAGACCGCTGCCCGAGTCGGAGCTCAAGCGCCAGGTCTGGCTGCTCTTCGAGTACCCCGAGAGCTCGAGTGCG
GCCCACCTCGTTGCGATTCTCTCCGTTGTCGTCATCTTGGTGTCAATTTTCATCTTCTGCATGGAAACGCTGCCG
CAGTTCCGAGACCATGCTGATCAAGAAGAGCAGTTCAATGCCACCGAAATTCCCTCAACCGCCAGTTTTGCCACG
GATCCATTTTTTCTGGTGGAAACCCTTTGCATCGTGTGGTTTTCCTTCGAGCTTGTGGCACGATTCCTCTCGTGC
CCTAGCAAAACTGCGTTCTTCCAAAACATCATGAACAGCATCGACGTGGTGGCCATCATGCCGTACTTCATCACG
CTCGGAATGGAGCTGGCCGAGCAGCAGGGCAACGGTCAGCAGACCATGTCGCTCGCCATCCTGCGCGTCATCCGC
CTGGTGCGCGTCTTCCGGATCTTCAAGCTGTCGCGCCACTCCAAGGGCCTCCAGATCCTGGGCCAGACGCTCAAG
GCGAGCATGCGCGAGCTGGGGCTGCTCATCTTCTTCCTCTTCATCGGCGTCATCCTCTTCTCGAGCGCCGCCTAC
TTCGCCGAGGTGGACGAGCCCAAGACGTACTTCACGAGCATCCCGCACGCGTTCTGGTGGGCCGTGGTCACCATG
ACGACGGTTGGCTACGGCGACAAGGTGCCCGTGACGGTGGGCGGCAAGGCGGTGGGGTCGCTGTGCGCCATCGCG
GGCGTGCTCACCATCGCGCTGCCCGTGCCCGTAATCGTGTCCAACTTCAACTACTTCTACCACCGCGAGACCGAC
GTCGACGACTCGCACTCTGGCAGGGCCCACTCTCCGGAGCACTCTGCTGCCAGGATGTCAATCGTCCAGTCGCTG
AGGAGGAGCGCGCCGAGCACACCGGTCCACGCACCAGGCTCCATGAGCAAGAGCGACAACACGCGCACACACACA
TATGTGGCCAACAGCTTTGAGCATCAGTGCAAGCCCACCGATGTGtga

KCNA_C Contig6469.4 (complement 5992-4469)
ATGACGGTGGTGGGTGTCAAGGCCGGCTCGGATGATGCGGCCGTGGCGGTGCAGGGAGGCTACCCGCAGGAGCGG
TACGAGCTGGCACGGCCCGGCGGGCACGAATTCTGCGAGCGCGTGGTCATCAACGTGTCCGGCCTGCGCTTCGAG
ACGCAGCTGCGCACGCTGGCCCAGTTCCCCGACACGCTGCTGGGAGACCCCCAGAAGCGGATGCGCTACTTCGAC
CCGCTGCGCAACGAGTACTTCTTCGACCGCAACCGGCCGAGCTTCGACGCCATCCTCTACTACTACCAGTCAGGC
GGCAGGCTGCGCAGGCCCGTGAGCGTGCCCTACGACATCTTTACGGAAGAGGTGCGCTTCTACGACCTGGGCGAG
GAGACGATGCTGCGCTATCGCGAGGACGAGGGCTACGTGAAGGAGCCCGAGAAGCCGTTGCCAGAAAATGAGTTC
CAGCGCCAGGTGTGGCTCCTCTTCGAGTACCCGGAGAGCTCCAGTCCAGCGCGTGCCATCGCCATCGTGTCCGTG
CTCGTCATCCTCATCTCCATCATCATCTTCTGTCTGGAGACGCTGCCCGAGTTCCGCGACGATGCAGACCTCTAC
AAGGTGTCGTACTACACGAACGGAACGCACTACTTCTACCACAACGTCTTTGCCGACCCCTTCTTCATCATGGAG
ACCATCTGCATCATTTGGTTTTCCTTTGAGCTCATGGTCCGCTTCTGCGCGTGCCCCAGCAAGGTTGAGTTCGGC
AAGAACCTAATGAACATCATCGACATTGTGGCCATCGTGCCCTACTTCATCACGCTCGGCACGGAGATGGCCGAG
CAGACTGGCAGTGCCAACGGGCAGCAGACGATGTCGCTCGCCATCCTCAGGGTCATCCGGCTGGTGCGCGTCTTC
AGGATCTTCAAGCTATCACGCCACTCCAAGGGGCTGCAGATCCTGGGCCATACGCTGCATGCGTCGATGCGCGAG
CTGGGCCTCCTCATCTTCTTCCTCTTCATCGGCGTCATCCTCTTCTCGAGCGCCGTCTTCTTCGCCGAGGTGGAC
AGCAAGAAGACGGAATTCCAAAGCATCCCCGACGCGTTCTGGTGGTCATTGGTGACGATGACCACGGTGGGCTAC
GGCGACATGTCGCCCAAGACGGTGGGAGGCAAGATCGTCGGCTCGCTCTGCGCCATCGCCGGCGTGCTCACGGTC
TCGCTGCCCGTGCCGGTCATCGTGTCCAACTTCAACTACTTCTACCAGCGCGAGACGGAAGGCGAGGACCAGACG
GTCTATTCGCACGTGCACAGCACGCCCTTCCACGAAGCGACGCACGCCGACAATGCCGAGGCCAACTCGGAGCTG

TGCCGCCGCAGCATTTGCAGCTTCTCGTCGTTCGAGAAGATCGACTGCAAGGAGGCGGACGAGCACGCGGACGAG
GACTCATGCGTCGAGGGCGAGCTCAAGCTCAGCAACTGCAACGTGGCGACGGCGCAGCAGCACAAGCACGTCGGC
GGCAGTCGGATCGAGACGGACGTGtga

KCNA_D (assembly after identity with a *Lampetra* EST (NY0AAB158YC06RM1) that spans the underlined section). Join Contig39975.1 (complement 504-71), Contig4036.1 (471-1243: a "C" deleted at pos 1238), Contig4036.2 (1368-1808)

ATGGAACACAGCTCGCCGGTAGCAGGCGCAGCACGAGGTCGCCTGAGCCTCGTGCGCGCCGAGGGCGACGGCGCG
TGGATGACCGCGGGACTCAAGTGCGACGCGGCTCTCAAGCAGAGCTGCTTCGAGACGCTGGCGTTGGACAGCAGC
GTCAGCGGTGGCGGCAGTGGCGCGGAGGCGCGGATGGGCGACCGCGTGTGCATCAACGTGTCGGGCGCGCGCTTC
GAGACGCGGCGGGCCACGCTGGCCCGGTTCCCGGCCACGCTCCTCGGGAATGCGCACCGTCTTGCGCGCCACTAC
GACGCGCAGCAAAACGAGTACTTCTTCGAGCGACACCGCGACAGCTTCGCCGCCATCCTGCATTTCTACCAGTCG
AGCGGCAGGCTCCAGCGTCCCACGATGGTGCCCATCGACATCTTCTGGGATGAAATCAGGTACTTTGACCTGGGA
GACAAGGTGTTGTCACAGTTGCGTGAAGAAGGCATGGATCCCACAGAGGACCTGGTGAACCCAAACGCCATG
CCGGTCCACACACGTGGTTTCCACAGCCACCTTTGGCTTCTCTTCGAGTCGCCCGAGAGCTCCAATGCCGCGCGG
GTCATCGCCATCGTCTCCGTGTTCGTGATCGTCGTCTCGATCGTCATCTTCTGCTTGGAGACGTTGCCCGAATTC
CGCGAGGAAGAAACCCATTTCAAGCAAGGGCTTGCCGGCAACACCACCGACACGACAAATGTCGTAGATCCATTC
TTCCTGGTGGAGACCACGTGCATGGTGTGGTTCTCGTTCGAGTTCATCGTCAGGCTCATTGCCTGTCCGTGCAAA
CCCACGTTCATCCGTGACCTCATGAACGTCATCGACCTCATCGCTATCCTGCCGTACTTCATCACCCTCATCACC
GACTTGACGGGGTCCATGGTGCCGGAGTACGTCTCGGAGGCGGCGACCACCACAAACGCGTACAGCTCAGTGGCC
ACCTCCTTGGCGACCGCGAGCCCAGGCACGGGGGACAGCATCAGCGAGATCGTTCCAGCCGGTGGCCTTCCCAGC
GTCACCGGGAACAACGGCCGTGGTGGCACAGTGACGGCCGGGACCGGGAGCAGTGGCAGTGGGGGGCAGACCATG
TCCCTCGCCATCATTCGCGTGGTGCGTCTCGTGCGCGTCTTCCGCATATTCAAGCTGTCGCGCCACTCCAAGGGC
CTCCAGATCCTGGGTCAAACACTGAAGGCCAGCATGCGGGAGCTGGGACTCCTCATCTTTTTCTCTTCATCGGA
GTCATCCTCTTCTCCAGTGCTGCCTACTTTGTGGAGGCCGATGACCCCGACTCGCACTTCACGAGCATCCCAGAT
GCCTTCTGGTGGGCCGTTGTGACGATGACCACGGTGGGCTACGGTGACATGCGGCCCATCACACTGGGAGGAAAG
ATTGTTGGCTCTCTGTGTGCCATCGCGGGCGTGCTCACGGTGGCACTGCCCGTTCCTGTTATTGTGTCCAACTTC
AACTTCTTCTACCAGCGAGAGCGCGATAGGCCTGAAGCCAAGGAGTCATTTTTGCTGGAGTTGGATGACACTCAC
ATGGACGCATCAGATCAAGAAGATGGAGACAGTGAATACGGGGTGTACGAGAATCACGTGGAGACAGATTTTtga

KCNA_E Contig8849.3 (complement 3020-1695)

ATGCGCGTAGATGAGGGGGATGCCCTCGCTTCGCGAGGTGGCGAGGGAACGGGGGCCGTGCCTGCGACCTTCGGC
CGCTGCAAGCCAAAGGTGAAGCACAAAGCGGCATCGCGTGCCAATGGGGCCAGTCCGCTCGCCCGTCTGCGGAAT
GAGGGTGCGGTCACACCTCCGCGTGGTGATTTGCAGCGAGTGGTAATAAATGTGTCCGGAGAACGGTTTGAGACG
CAGTTGAAGACGCTGGACCAGTTCCCAGACTCGCTCCTGGGCCACCCGGACAAGCGAGCGCGATATTTTGACCCA
CTCCAAAACGAGTACTTTTTTGACAGAAGCCACGTCAATTTTGATGCCATCCTCTTCTACTACCAGTCTGGGGGA
AGGCTTTGCAAGCCTGTTGACGTGCCTTCGGATGTCTTCAGAGAGGAGGTAGAATTCTACGAACTCGGAGAAGAA
GCCGTTGCAAAGTACGGAGAGAATACACTCGACACCCCTGAAGAGGAAAACGTGCACGGAAAAGACTTTCAGCAG
CACGTATGGCGCTTGTTTGAATCGCGAGACGGTTCCATCGCTGCAAAGATGTTATCCCTCATGTCGTTTGTGACG
ACTATACTGTCGATTACGGTCATGTGTTTGGAGACACTGCCACAATTTCGCAAAGATAATCAAGTCCATGCCAAT
CGTACCGAGTTCAGCATTCGAACAAGTATCTACAATCTCGATCCTTTCTTCATGTTGGAAACGGCGTGTGTTGTT
TGGTTCATATTTGAGCTTGCCGTTCGCTTTTACGCCTGTCCAAGCAAGACGTCCTTTATGAAGGATTTCATGAAT
GCGATCGACATCCTGGCAATAATGCCATATTTCATCACGGTTGTCTTGGAGATCATGCAACAGCAAGATCAGACC
CGGCAACCTTTGCTGAAGATCATACGCCTCGTAAGGGTCTTGCGAATATTCAAACTGTCTCGTCACTGCCAAGAT
CTGCAAATCCTTGGGCTGACGCTGAAGGCCAGCCTGCGGGAGTTGGCCATGCTAAGCTTTTTCCTCTTCGTCGCT
GTGGTAATATTCTCCGGTGCGATCTACTACGTGGAAGCTGACGACCCGCAGAAACACTTCAGCAGCATCCCAGAC
GCCTTCTGGTGGGCGGTGGTCACAATGACGTTGGTTGGCTACGGAGATATGTGGCCCATGACGGTGGGAGGCAAG
ATCGTGGGTGCGCTATGTGCTATCACTGGAGTGCTCACCATCTCTCTACCAGTACCCGTCATTGTTTCGAACTTC
AAATACTTCTATAATCTAAAAAACAAGGACATTAAGAGATGTCGTCGTTGCtaa

KCNA_F Contig109.11 (3418-5049)

ATGCCCGCCGTTCGAGGAGAACTGTTCTCGCTCGACGAGAAGGACCTACTGCTGCCCACGAATTGGCCAGCTTTG
GCCGACCCGACGGACGACACGCCGCCAACAAAGGAGTCCGGTGTCCTGGCCGGCGACCGCCGCCGCGAGAGGATC
GTCGTCAACGTGTCGGGCCTGCGCTTCGAGACGGAGCGGCGTACCCTCGAGCGCTACCCCGGCACCCTGCTGGGC
GACGCGTCACGTCGCGCCCCGTTCTACAGGCCCGACCTCGGCGAGTTCTTCTTCGATCGCCACCGTTCAAGCTTC
GAGTCGGTGCTCCACTTCTACCAGTCGGGCGGAGAGCTGAGACGGCCGTGCGCCGTGCCCCCGCTCACCTTCGTC
GGCGAGGTGCTCTTCTTCGACCTGGGCGACGAGGCCGTGGAGAGGCTCAGGGAGGAGGAGGGCTTAGCGGACGAC
GACGAGGTGGAGGAGGCGACGCTGAAGGCTGAGAGGAAGATGGCGAAAAAGGAGGCTCAGCAGCGGCTGCCTTCG
GGGAAGGTGCGCCGCTACCTGTGGAATCTATTCGAGCACCCGGACACGTCCACGTCCGCCAAGGCCACGGCCGTC
TTCTCCTTGGTCATGGTGCTGCTGTCCGTGGTCACGTTCTGCGCGGAGACCCTGCCCACGTTCAGGAACAGGCAC
CACCGGCGGCACCAGCACGGCCGCAACGACTCCGCGGACTCGCAGCCGAGCTCCGAGTGGATGGACCCCTTCTCG
GACCCGCTTTTCCTGGTGGAGACCGTGTGCATCGCCTGGTTCGGCCTGGAACTCCTCATCCGCGCCTTCGCGTGC

CCGAACAAGCTGGCGTTCGCACGCGACCCCATGAACATCGTCGACTTCATCTCCATCCTGCCCTACGGCATCACC
CTGGCCATGGACCAGCTCGGCTCGTCGGCCACCGGCGAGAAGAGCAAGGCGAGCAACCTGGCCGTGCTCAGGACC
ATGCGGCTGCTGCGCGTGTGCCGCATTCTCAAGCTGTCGCGCCACAGCCAGGCCCTGCAGGTGCTGGCGAAGACG
CTGCGCGCCAGCTTCCAAGAGATCGTCACCCTGCTCATCTTCGTCGCCATCGCGGTCGTGTTGTTCGCGAGCGCT
ATCTACTTCGCCGAGGTGGGCGCGGACGAGGCGCACTTCAACAGCGTGCCCGACGCCTTCTGGTGGGCCGTGGTG
ACGCTCACCACGGTGGGCTACGGGGACATGACCCCGGTGACCCTGCCCGGCAAGCTGGTGGGCTCCATGTGCGCA
TTGACCGGGGTGCTCCTCATTGCCCTGCCCGTGCCCGTGATCGCCAACAACTTCACCACCATCTACGAGCTCCAG
AAGAAGAAGATGATGATGATGAAGAAGAAGAAGAAGAAGAAGACGATGATGACGACTAAGAAGGGGACGTTGAAT
GTCATGAACAACGAGGCGCCGCACGTCACCGACGGCCACCAGGCGCATGGCCCGGTGGGGTCATCCGAGAGGTCC
CAGCAGAGGTGCTCGCCGTGTGGGGTCCTTTGCTGCGTGCATGGGCCGCGCCGGTGGTGCGACACGGAGGAGAAG
CTGGGACCTCGAGAGCGGCAGCAGAATGAGCTTCGATACCCACATGAGACCAAAGTGtga

KCNA_G Join Contig16470.1 (complement 379-1) trace [GenBank ti|1427225648] (complement 498-1)
ATGTCCAGCGTCAGTGTCGCGGAGCTCACCATGAGACGCGACTGCGCTTCGCCACAACCCGCCACGGACAGCAGC
TACACCACCCTCTTCACCACGGAGGACCTGCCGAACAATCACCACCACCACCAGTGTCCCCAACAACAACAGCAT
CAGCAGCAGCATCAGCAGCATCAGCAGGGCCAGGAGCTGCCGGTCGCGGCTTGGAGCGAGCGAGTGGCCATCAAC
GTGTCGGGCCTCAGGTTCGAGACGCGGCGCAGCACACTGGAGCGTTTCCCCCGCACGCTCCTGGGCGACGGCGCT
CGCCTGCTGCGCTTCTTCGACCCGCTGCGCGACGAGTACTTCTTCGACCGCAACCGCGACAGCTTTGGCGCGATC
CTCTTCTACTTCCAGTCGGGCGGCAGCCTCCACCGGCCCTCCGCGGTGCCCCACGGCCTCTTCGTGCAGGAGGTG
CGCTTCTACGACCTGGGCGCCGAGGCGCGCGGCCGCCTCTGCGAGGAGGAGCGCTGTTCCCGCGAGGACCCCGAG
AGGCCGCCCCTGCCGCGCAACCGCCTGCAGCGGCGCGCCTGGCTACTCTTCGAGCACCCCGAGAGCTCCGTGTGG
GCGCGCGCGGTCGCCCTGGTCTCGGTGCTCGCGACGCTCGCGTCCGTGGTGGTCTTCTGCCTCGAGACGGTGCCG
TACGGCGAGCGGAAGAGCGGCGGCAGGAAGGGCAGCGGCGAGGGGGAGGAGGGGGAGGAGGGTGGTGGTGGTGGT
GGTGGTGCGGCGGCGGCCGCGTTCGCCGTCGAGGTGGCGTGCATCGCCTGGTTCTGCCTCGAATTCCACCACATT
GGAGCTTCAGCTGCTCGAGATGGTCATAGCTGTTCCTGCGTATGTGTATTAC???

KCNA_H Contig22917.3 (18-1490)
???GAGCGCGTGGTGGTGAACGTCTCCGGCCTGCGCTTCGAGACGCAGCTGCGCACGCTCGCGCACTTCCCCGAC
ACGCTGCTGGGCGACCCCCAGAAGCGGATGCGCTACTTCGACCCGCTGCGCAACGAGTACTTCTTCGACCGCAAC
CGACCGAGCTTCGACGCCATCCTCTACTACTACCAATCGCGCGGCAAACTGCGCAGGCCCACCAACGTGCCCTAC
GACATCTTTGCCGAGGAGGTGCGCTTCTACGAGCTCGGCGAGGAGACGATGGCGCGCTACCGCGAGGACGAGGGC
TACGTGAAGGAGGAGGAGAAGCCGCTGCCCGAGAACGCGCTCCAGCGCCAGGTGTGGCTCCTCTTCGAGTACCCG
GAGAGCTCGAACCCCGCGCGCGTCATCGCCATCGTGTCCGTGCTCGTCATCGTCATCTCCATCGTGATCTTCGTG
GCCGAGACCCTGCCCGAGTTCCGCGACGACAAGGACCTCCACTCGGGCACGCGCCACGACGTCGGTGCCGGCGAC
GCCGCTGTTGCCGCTGCTGGCAACAGCAGCGGCGGCAGCAGCGGCGGCGGCAGCGGCCACGCGCACGGAGGTCAC
GCGCACCCGGGCGGCGGCGGCGGTGGCGGCGGCGCGCTCGGCCACGGTTACGGCCACGTGCCGGGCGTGCACGTG
GCTGTGCCGCACGGCCGCGGGAGGCCCACCCCCGAGTTCGGCAAAGACCCGTTCTTCATCGTGGAGACCGTGTGC
ATCGTGTGGTTCAGCTTCGAGCTGCTCGTGCGCTTCTGCGCGTGCCCCAGCAAGCCGGGCTTCTTCAAGAACATC
ATGAACACCATCGACATCGTGGCCATCATGCCCTACTTCATCACGCTGGGCACGGAGCTCGCGGCGCAAGACACG
GGCGCCAACAACGGGCAGCAGACGATGTCGCTCGCCATCCTCAGGGTCATCCGGCTGGTGCGCGTCTTCAGGATC
TTCAAGCTGTCGCGCCACTCCAAGGGGCTGCAGATCCTGGGCCAGACGCTGCGCGCGTCGATGCGCGAGCTGGGC
CTCCTCATCTTCTTCCTCTTCATCGGCGTCATCCTCTTCTCGAGCGCGATTTACTTCGCCGAGGTGGACGACCCC
AACACGCACTTCCACAGCATCCCCGAGGCCTTCTGGTGGTCGCTCGTCACCATGACCACGGTGGGGTACGGAGAC
ATGTTCCCCGTGACGCTGGGCGGCAAGATCGTGGGCTCGCTGTGCGCCATCGCGGGCGTGCTCACCATCGCTCTG
CCCGTGCCCGTCATCGTGTCCAACTTCAACTACTTCTACCACCGCGGCTCGGAGGCCGAAGAGCAGACGCTGTCT
GTGCAGGAGGACAGCGGCGGCAGCTGCGAGGCCGGCAACCAGATCAAGAGGAGTAACAGCGTCTCGTCCGTCAGC
AAGTCCGAGTACATGGACGACATGGCCTTCCTCGGACCCAACTACATCGAGtga

KCNA_I Contig25309.7 (2nd exon: complement 1912-1295)
agACCATGTCGCTCGCCATCCTGCGCGTCATCCGCCTGGTGCGCGTCTTCCGGATCTTCAAGCTGTCGCGCCACT
CCAAGGGCCTCCAGATCCTGGGCCAGACGCTCAAGGCGAGCATGCGCGAGCTGGGGCTGCTCATCTTCTTCCTCT
TCATCGGCGTCATCCTCTTCTCGAGCGCCGCCTACTTCGCCGAGGTGGACGAGCCCACGACGTACTTCACGAGCA
TCCCGCACGCGTTCTGGTGGGCCGTGGTCACCATGACGACGGTTGGCTACGGCGACACGGTGCCCGTGACGGTGG
GCGGCAAGGTGGTGGGGTCGCTGTGCGCCATCGCGGGCGTGCTCACCATCGCGCTGCCCGTGCCCGTCATCGTGT
CCAACTTCAACTACTTCTACCACCGCGAGACCGACGTCGACGACTCGCACTCCAACGTAGCGCAGTCGCCGAAAA
ACTCCGCAGTTAATTACCCCTCGAATGAGTCGCTGAAGAAGAGCGCGCCAAGCACACCGGTGCACACGCCGGGCT
CCGTGAGCTGCCACTCCGTGAGCAAAGGCGACAACACGCACACACACACGTACGTGGCCAACAGCTATGAACATA
AGTGCCAGCCCACCGACGTGtga

KCNA_J Contig78404.1 (complement 1594-980)
???CAGCACGCCATGTCGCTCGCCATCCTCAGGGTCATCCGCCTCGTGCGAGTCTTCAGGATCTTCAAGCTGTCG
CGCCACTCCAAGGGTCTGCAGATCCTGGGCCAGACTCTGCGCGCCTCCATGCGCGAGCTGGGCCTGCTCATCTTC

TTCCTCCTCATCGGCGTCATCCTCTTCTCGAGCGCCGTCTTCTTCGCCGAGGCGGACAGCGACGAGTCGCACTTC
ACGAGCATCCCCGACGCCTTCTGGTGGGCCGTGGTCACCATGACCACGGTGGGTTACGGCGACATGAGGCCCGTC
ACAATCGGCGGGAAGATCGTGGGCTCGCTGTGCGCCATCGCGGGCGTGCTCACCATCGCGCTGCCCGTGCCCGTG
ATCGTCTCCAACTTTAACTACTTCTACCACCGCGAGACGGACAATGACGACTCGGCGGTCAAAGTCCAGGGCGGC
GGTGGTGGCGGTGGTGGCGCCGGTAGCGGTGGCGGCTCCTCTGGTGCCGTAGGGCCGCGTTCGTCGCAGGGGAGT
GGAACGCCGAGGAAGCGCCGCCCCAGCAGCGCCCAGGGCCTCGACGCCGAGACGGACTCGAGCACGGAGTCGGGA
GATCCAAACTCCTCGGGA???

KCNA_K Contig46381.2 (1-519)
???GGGCCTCTTCATCTTCTTCCTGTCATCGGGGTCATCCTCTTTGGAGCGCCGATCTACTTCACCGAGTCCGAC
GATTCCGACACGGACTTCACGAGCATCCCGCACGCCTTCTGGTGGGCCGTCGTCACGATGACCACGGTGGGCTAC
GGCGACATGTGGCCCAAGACCGTGGGCGGGAAGATCGTGGGCTCGCTGTGCGCCATCGCGGGCGTGCTCACGATC
GCGCTGCCCGTGCCCGTGATCGTCTCCAACTTCAACTACTTCTACCACCGCGAGATGGACAGCGAGGACCAGGGT
GAGTACTCGCACGTGTCCAGCCTCAACACCAGTGACGACGGAGGAGGTGGAGGAGGGGGAGGAGGTGGAGGCGAT
GAAGAAGTCTTCAAGCAAATGTCCATGGAGGTCAACCCGCAGAGCGGCAGGGGCGATGGGGCTGACGTGGTGTCC
ATCCTCAAGGAGGTGGACTCCGGACAAGTGCCCAATAGCCTGAGTGAGCAGACGGGGCTAAGACCCATTGCCtag

KCNA_L [GenBank:ti|1291002197] (complement 813-1)(No hit in contigs)
???TGCGCTTCGAGACGCAGCTGGGCACGCTCGCCAAGTTCCCCGACACGCTGCTGGGCGACGCGGAGAGGCGGT
CGTGCTACTTCGACCCCCTGCGCAACGAGTACTTCTTCGACCGCAACCGACCCAGCTTCGACGCCATCCTCTACT
ACTACCAGTCGGGCGGCAGGCTGCGCAGGCCGGCGAGCGTCCCCTTCGACATCTTCGCCGAGGAGATCAAGTTCT
ACGAGCTGGGCGACGAGGCGATGGTGCGCTTCCGCGAGGACGAGGGCTACGCCAGGGAGGAGGAGAGGCCCATGC
CGGCGAGCGAGTTCCAGCGCCGCGTGTGGCTGCTCTTCGAGTACCCCGAGAGCTCGGGGGCGGCGCGCGGCATCG
CCATCGTGTCGGTGCTCATCATCGTCGTCTCCATCATCATCTTCTGCGTCGAGACGCTGCCCATGTTCCGGGAGG
AGAGGGAGAACACCTCGCTCGCCGTGCCATTCGTCAACGGCACTGAGGTGGAGGGCGCGGCCTCCGGCAGCTCGT
TCATGGACCCGTTCTTCATCATCGAGACCATCTGCATCGTGTGGTTCAGTTTCGAGTTCACGGTGCGCTTTCTCG
CAAGTCCCAGCAAGCCGGCCTTCTTCAAGGACATCATGAACATCATCGACATCGTGGCCATCGTGCCTTACTTCA
TCACCCTGGGCACTGAGCTGGCGGAGCAGCAGGGCAACGGGCAGCAGACGATGTCGCTCGCGATCTTCAGGGGAA
TTCCACCACATTGGAGCTTCAGCTGCTCGAGATGGTCATAGCTGTTCCTGCGTATGTGTAGAAAAG???

KCNA_M 1st exon: join [GenBank:ti|1427218636] (54-741) [GenBank ti|1449626312] (599-817) (No hit in contigs)
ATGTGGTGGAATTCCGGCGGCGGCGGCGGCGGAGGAGGTCCCGGGGGGTCGCGAGGATTCGGCGGCTGCCGCGCC
CCGCACGGCCACGCGGTGCTGACGCCGCACGCGCTCGACGTCCTGCTGCTGGACGCGGCTGCGTGCGAGCCCTAC
TACCAGCACTACAACAACCACCACCACCACCACCACAACAATCTGCAGCAGCAACAACCGCAGCAGCAGCCGCAA
CAGCAGCACAACAACCATCACAACCACCATCACCAGCAGCATCGCGGCAGCAGCAGCGGCGGCAGCAGCAGCAGC
TTCAGCGCCGGCGGCGGCGTCGACTCGGGCGGGTTCCCGGAGGCGGGCGAGCGGCTCACCATCAACGTGTCGGGG
CTGCGCTTCGAGACGCACGCGCGCACCCTGGCGCGCTTCCCGCGCACGCTGCTGGGCGACCCGCGCAAGCGAGCG
CCCTACTTCGACGCGCTGCGCAACGAGTTCTTCTTCGACCGCAACCGCCAGAGCTTCGACTCCATCCTGCACTAC
TACCAGTCACGCGGGCGCCTGCGCAGGCCCGCGGGCGTGCCCGTGGACGTGTTCGCCGAAGAGCTGCGCTTCTAC
GAGCTGGGCGACGAGGCGATGGCGCGCTACCGCGAGGACGAGGGTTGCGCGCCCGAGGAGGCCGAGCGAGTCCTG
CCGCGCGCGCCCCTGGCCCGCCAGCTGTGGCTCCTCTTCGAGTACCCCGAGAGCTCGGCGCCCGCGCGCGCCATC
GCCATCGTGTCGGTGGCGGTCATCGTCCTCTCCATTATCGTCTTCTGCCTCGAGACGCTGCCGCAGTTCCGAGAC
GAGAGGCGCGACGCCGCCGCCGCATACAACAACGATAACACAATAATAACGACAACTACAACAACAACCTGTGCA
TCAGCACgt

KCNA_N join [GenBank:ti|1489701441] (complement 502-22) [GenBank ti|1223432487] (247-1)(No hit in contigs)
???GCCTCTCTGACGACCCGTTCTTCGCCGTGGAGTCGCTGTGCGTGGTGTGGTTCAGCGTCGAGCTGCTGGCAC
GCTTCCTCGCCTGCCCGGGCAAGGCGGCCTTCTTCCGCGACGTCATGAACACCATCGACGTCGTCGCCATCGTGC
CGTACTTCGTCACGCTGGGCACCGAGCTGGCTGAGCGAGGCGGTGGAGGCGGCGGCGGAGGCGGTGGAGGCGGTG
GAGGCGGCGGAGGCGGCGGAAGCGGCGGAGGCGGCGGCGGAGGCGGCGGAGGCGGCGGCGGAGGCGGTGGAGGCG
GCGGGGCTCAGACCACGTCGCTCGCGACCCTGCGCGTCATCCGCCTCGTGCGCGTCTTCCGCGTCTTCAAGCTCT
CGCGCCACTCCAAGGGCCTCCAGATCCTGGGCCAGACCCTCAGGGCGTCCATGCGCGAGCTGGGCCTGCTCATCT
TCTTCCTCTTCATCGGCGTCATCCTCTTCTCGAGCGCGGCCTACTTCGCCGAGTTCGACGAGCCCCGCACGTACT
TCCTGAGCATCCCGCACGCCTTCTGGTGGGCCGTCGTCACCATGACGACGGTGGGCTACGGCGACGCGGTGCCGG
CGACGACCGGCGGCAAGGTGGTGGGCTCGCTGTGCGCCATCGCCGGGGTGCTCACCATCGCCCTGCCCGTGCCCG
TCATCGTCAGCAACTTCAACCACTCTACCACCGAATCCACCACAGTGGCGAAAAC???

KCNA_O join [GenBank:ti|1443482662] (148-794) [GenBank:ti|1383256050] (complement 391-62)(No hit in contigs)
ATGGAGGGCGGAGAGCGTGGCAGGCGAGACGTCCACGGGCAGGGCCGCTGCGGCGGCTACGAGCGCGTGGTCATC
AACGTCTCGGGGCTGCGCTTCGAGACGCAGCTCGAGACGCTGGCGCATTTCCCGGACACGCTGCTCGGCGACTCG

GCGCGGAGGATGAACTTCTTCGACTTCCTGCGCAACGAGTACTTCTTCGACAGGAACCGGCCGAGCTTCGACGCG
ATCCTCTACTACTACCAGTCCAAGGGGAAGCTGCGGAGGCCCGACAGCGTCCCGTACGACATCTTCATCGAGGAG
GTCAAGTTCTACGAGCTCGGCGAGGAGACCATCACGCGCTACCGCGAGGACGAGGGCTACGCGAAGGAGGAGGAG
AAGCCGCTGCCCGAGAGCGCGTTCCAGCGCCAGGTGTGGCTCCTCTTCGAGTACCCGGAGAGCTCCAGCCCCGCG
CGCATCATCGCCATCGTGTCCGTGCTCGTCATCGTCATCTCCATCGTGATCTTCGTGGCGGAGACCCTCCCCGAG
TTCCGGGACGAGAAGGAGCTTCACTCGCGGCCGCGGCCCCTCGGCAACGGCACGGAGGCGCGGCCGGCGCCCTCC
TTCGTCACGGACCCCTTCTTCATCGTGGAGACGATCTGCATCATCTGGTTCTCGTTCGAGCTGCTCATCCGCTTC
TGCGCGTGCCCCAGCAAGCCGGGCTTCTTCAAGAACATCATGAACTCCATCGACATCGTGTCGATCATGCCCTAC
TTCATCACCCTGGGCACGGAGCTTGCCGAGCACACGGGCTCCGCGAACGGACAGCAGAGCATGTCGCTCGCCATC
TTCAGGGTGGTGCGTCTCGTGCGCGTCTTCAGGATCTTCAAGCTATCGCGCCACTCCAAGGGGCTGCAGATCCTC
GGCCAGACGCTGCACGCCTCCATGCGCGAGCTGGGTCTGCTCATCTTCTTTCTCCTCATCGGGGTCATCCTCTTC
TCA???
