# Supplementary material for: Emergence and evolution of the glycoprotein hormone and neurotrophin gene families in vertebrates
Source: BMC Evol Biol. 2011 Nov 15;11:332. doi: 10.1186/1471-2148-11-332 (PMC3280201; doi:10.1186/1471-2148-11-332)
Supplement: Additional file 8 — Human GPH-related gene paralogous gene sets and ancestral chordate and vertebrate proto-chromosome relics. Observed boundaries of the paralogous gene sets comprising the GPH subunit-related genes in human compared to segments (S) or conserved vertebrate linkages (CVL) predicted to derive from the two whole genome duplication events of, respectively, ancestral chordate proto-chromosomes (proto-chr.) G and D, according to [45] and of ancestral vertebrate proto-chromosomes 11 and 13/14 according to [44]. [GPHβ] tetra-paralogon seems to correspond to the sum of the segments derived from proto-chr. 13 (given in red) and 14. [file 1471-2148-11-332-S8.PDF]

Paralogous gene sets	Observed boundaries	Ancestral **chordate proto-chr. 13 and 14** derived segments		Ancestral **vertebrade proto-chro. D** derived linkage groups	
[LHβ]	Chr 19: 49-51, 56-59 M	S19.6	Chr 19: 49-59 M	CVL96	Chr 19: 49/55 M*
	Chr X: 47-56, 153-154	SX4/8	ChrX:47-56/151-155 M		
	Chr 7: 77-140 M	S7.6	Chr 7: 102-149 M	CVL41/44	Chr 7: 75-87/102-142 M
	Chr 10: 5-15 M	S10.1	Chr10: 0-15 M	CVL57	Chr 10: 5-15 M
[GPHβ-Ghost]	Chr 12: 0-124 M	S12.1/3-4/7-9	Chr 12: 0-6/16-47/67-107 M	CVL64/66	Chr 12: 0-6/17-45 M
		S12.4/6/8**	Chr 12: 32-47/56-67/90-99 M	CVL68	Chr 12: 68-105 M
				CVL106	Chr 20: 41-50 M
	Chr 22: 33-51 M	S22.3**	Chr 22: 42-49 M		
[FSHβ]	Chr 3: 0-15, 47-73, 125-129M	S3.1/3/6	Chr 3: 0-15/48-76/75-124 M	CVL14/18	Chr 3: 0-15/47-75 M
	Chr 11: 0-70 M	S11.1/3	Chr 11:0-3/8-62 M	CVL60/62	Chr 11: 0-60/68-72 M
[TSHβ]	Chr 1: 111-115/200-210 M	S1.8/10/15	Chr1: 102-111/112-115/199-210 M	CVL3/6	Chr 1: 110-115/195-205 M
	Chr 6: 35-42 M			CVL31	Chr 6: 34-38 M
Paralogous gene sets	Observed boundaries	Ancestral **chordate proto-chr. 11** derived segments		Ancestral **vertebrade proto-chr. G** derived linkage groups	
	Chr 1: 210-245 M	S1.16	Chr 1: 210-247 M	CVL7	Chr 1: 205-245 M
[GPHα]	Chr 2: 18-68 M	S2.2	Chr 2: 28-69 M	CVL9	Chr 2: 30-70 M
	Chr 20: 6-25 M	S20.3	Chr 20: 6-29 M	CVL100	Chr 20: 10-26 M
	Chr 6: 40-44/88/152/170 M	S6.4	Chr 6: 37-44 M		
[GPB5]	Chr 14: 27-106 M	S14.2	Chr 14: 29-106 M	CVL74	Chr 14: 25-105 M
	Chr 15: 33-43 M	S15.1	Chr 15: 0-41 M	CVL75	Chr 15: 28-42 M
[GPA2]	Chr 11: 61-67 M	S11.4	Chr 11: 62-66 M	CVL61	Chr 11: 62-67 M
[GP-Ghost]	Chr 19: 36-49 M	S19.5	Chr 19: 37-49 M*	CVL93/95	Chr 19: 37-44/45-48 M*

* coordonates according to Map viewer Ensembl release 62.

** S12.4/6/8 and S22.3 were indicated as potentially being derived from both proto-chr 13 and 14 [44]
